# Supplementary material for: Cardiac risk stratification in cancer patients: A longitudinal patient–patient network analysis
Source: PLoS Med. 2021 Aug 2;18(8):e1003736. doi: 10.1371/journal.pmed.1003736 (PMC8366997; doi:10.1371/journal.pmed.1003736)
Supplement: S1 Fig — The number of clusters represents different K values ranging from 3 to 10 in K-means clustering. The log-rank test was used to evaluate the statistical significance. All pairwise p-values between the subgroups for each K value were summarized in S2 Table. CVD, cardiovascular disease; KM, Kaplan–Meier. (PDF) [file pmed.1003736.s002.pdf]

S1 Fig

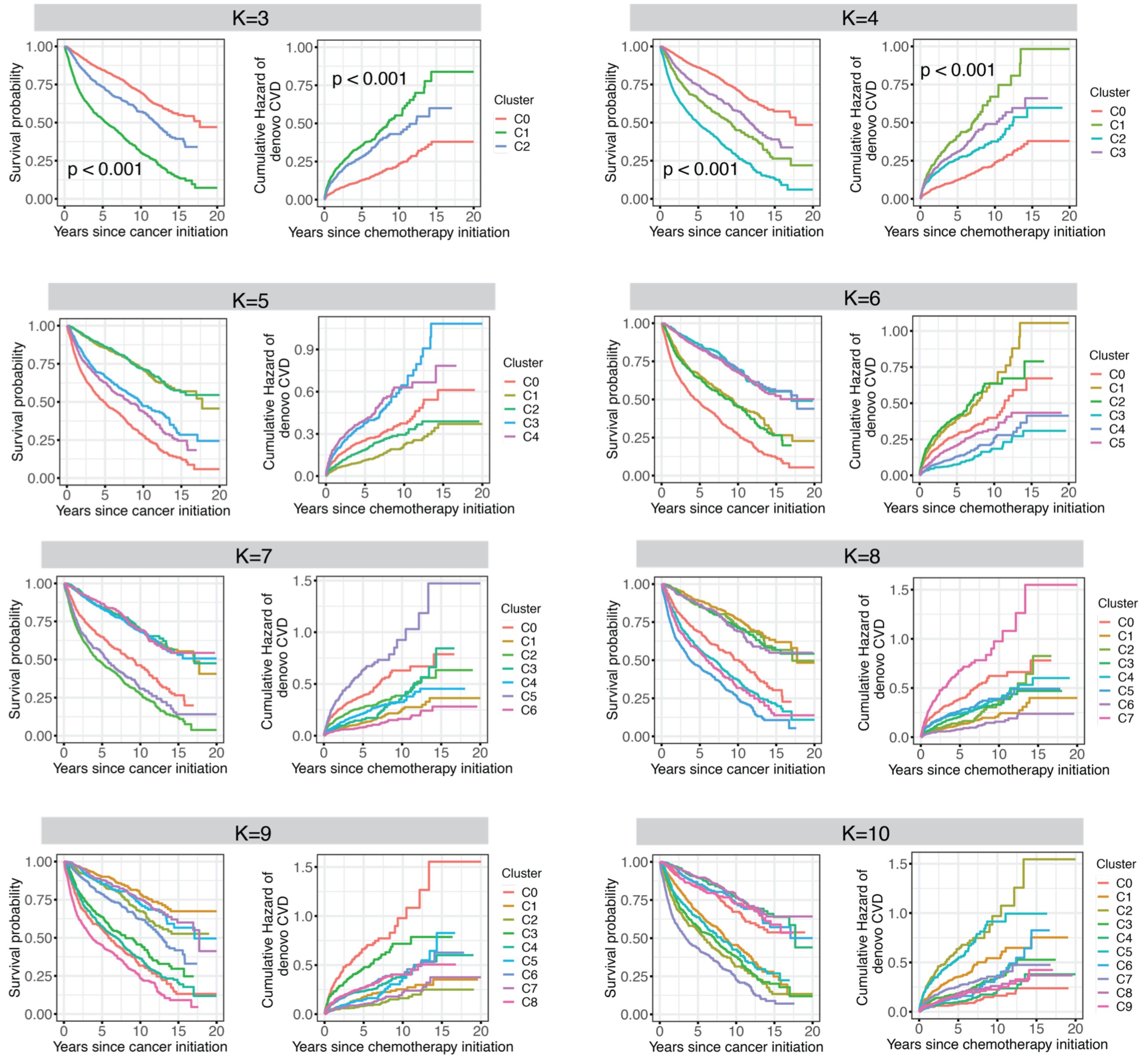

S1 Fig. Kaplan-Meier curves to estimate the survival and cardiovascular outcome for different number of clusters. The number of clusters represent different K value from 3 to 10 in K-mean clustering. The Log-rank test were used to evaluate the statistical significance. All pairwise p values between the subgroups for each K value were summarized in **S2 Table.**
