# Supplementary material for: Cardiac risk stratification in cancer patients: A longitudinal patient–patient network analysis
Source: PLoS Med. 2021 Aug 2;18(8):e1003736. doi: 10.1371/journal.pmed.1003736 (PMC8366997; doi:10.1371/journal.pmed.1003736)
Supplement: S2 Fig — (A) The workflow of K-means clustering stability test. (B) The ARI and AMI among the 100 repeats showed high stability of the clustering results. The averages and standard deviations are shown in the bar plot. AMI, adjusted mutual information; ARI, adjusted rand index. (PDF) [file pmed.1003736.s003.pdf]

S2 Fig

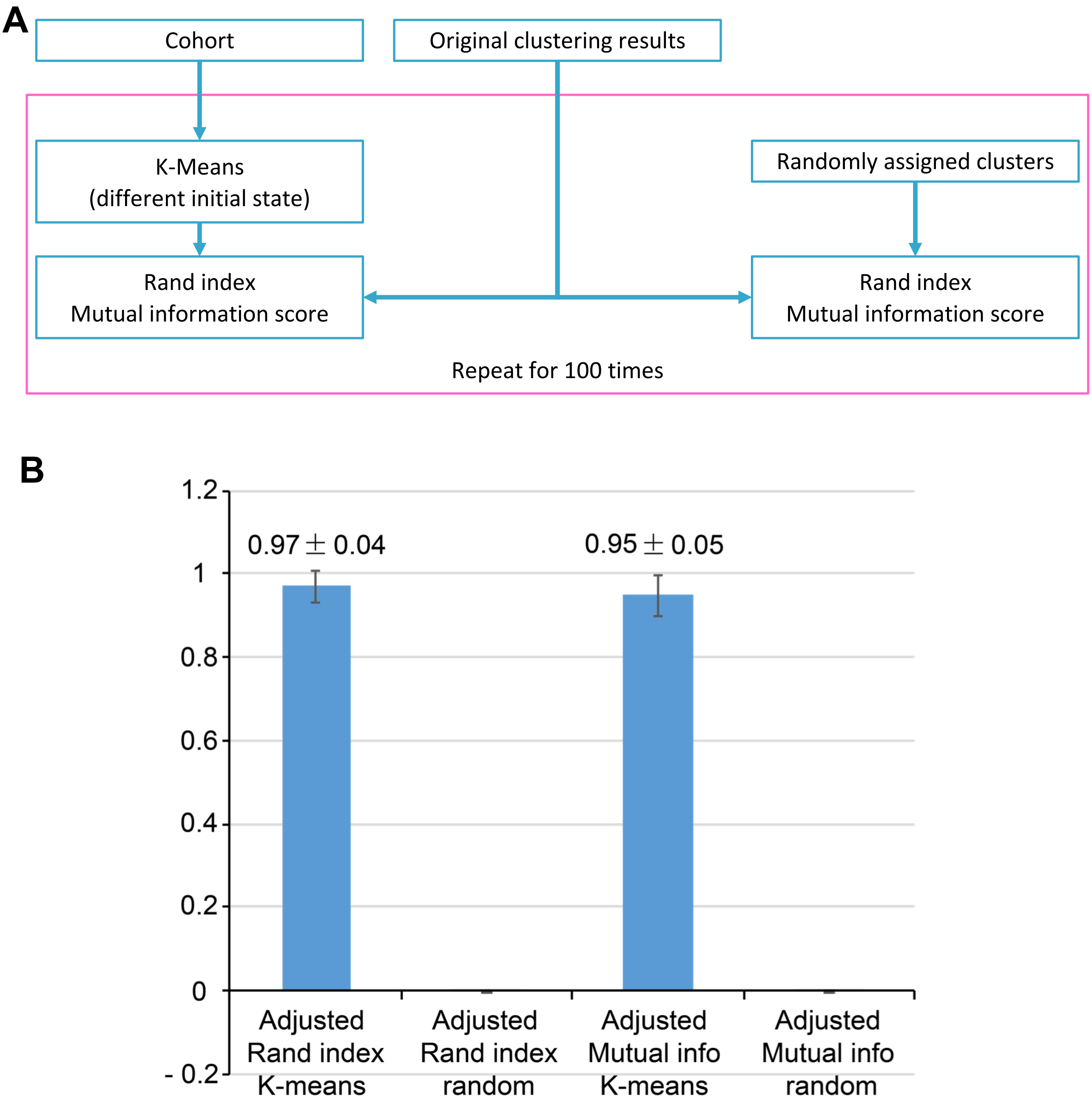

S2 Fig. Clustering stability test. (**A**) The workflow of K-means clustering stability test. (**B**) The adjusted rand index (ARI) and adjusted mutual information (AMI) among the 100 repeats showed high stability of the clustering results. The averages and standard deviations are shown in the bar plot.
