# Supplementary material for: Cardiac risk stratification in cancer patients: A longitudinal patient–patient network analysis
Source: PLoS Med. 2021 Aug 2;18(8):e1003736. doi: 10.1371/journal.pmed.1003736 (PMC8366997; doi:10.1371/journal.pmed.1003736)
Supplement: S3 Fig — The network density at different cutoff values and selected the cutoff that resulted in the lowest network density. Network density is defined as the ratio of the number of actual links and the number of all possible links from all the patients. (PDF) [file pmed.1003736.s004.pdf]

S3 Fig

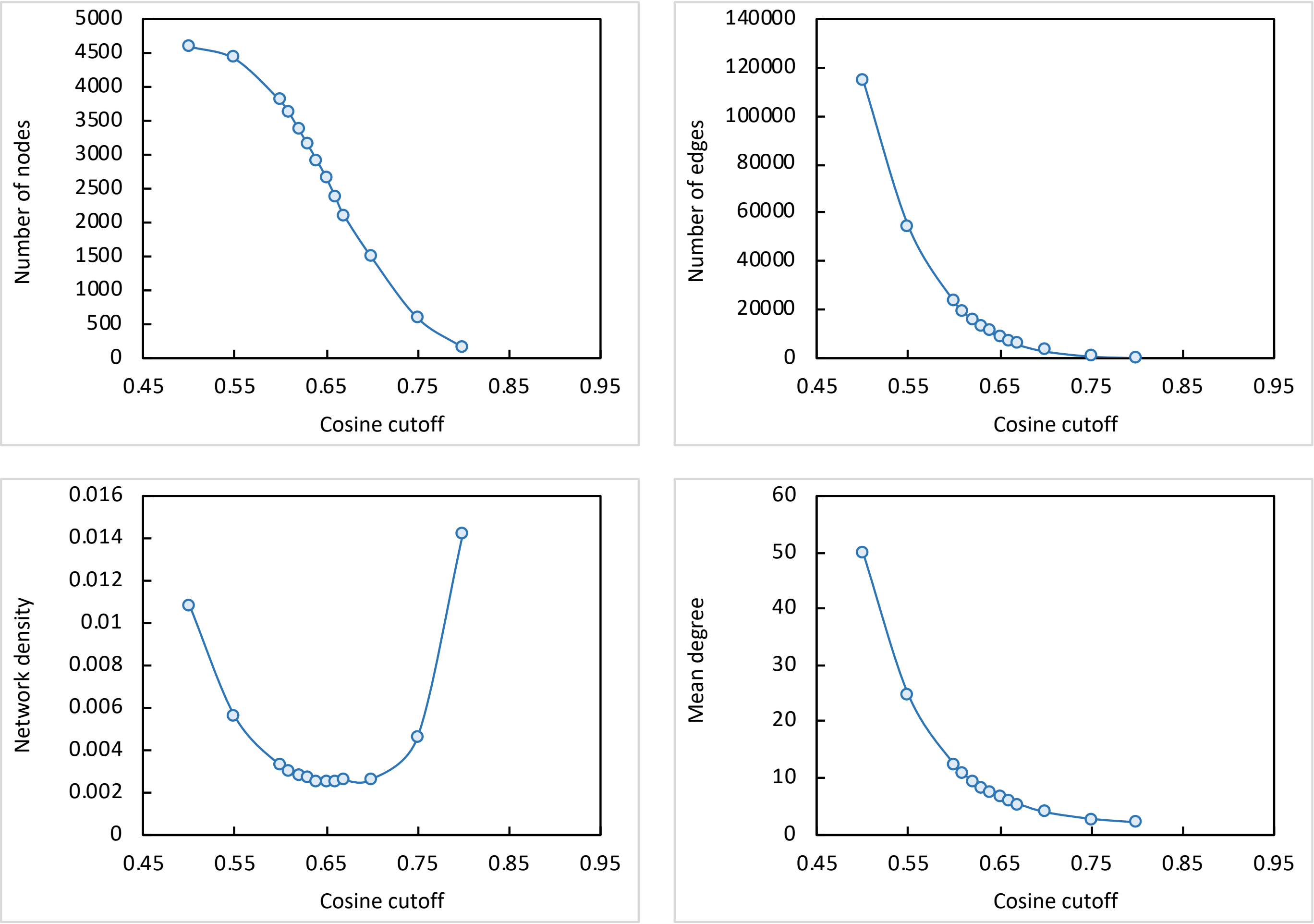

S3 Fig. Network density-based cosine cutoff selection. The network density at different cutoff values and selected the cutoff that resulted in the lowest network density. Network density is defined as the ratio of the number of actual links and the number of all possible links from all the patients.
