# Supplementary material for: Cardiac risk stratification in cancer patients: A longitudinal patient–patient network analysis
Source: PLoS Med. 2021 Aug 2;18(8):e1003736. doi: 10.1371/journal.pmed.1003736 (PMC8366997; doi:10.1371/journal.pmed.1003736)
Supplement: S4 Fig — (A) Patient–patient network colorized by 4 cluster numbers. All edges have PCC < 0.65 for the patient pairs. All data preprocessing and PCC cutoff selection were same with the method cosine similarity calculation. The network was visualized using Cytoscape v 3.7.1. (B) KM curves to estimate the all-cause survival probability in the 4 subgroups. The log-rank test was used to evaluate the statistical significance. KM, Kaplan–Meier; PCC, Pearson correlation coefficient. (PDF) [file pmed.1003736.s005.pdf]

S4 Fig

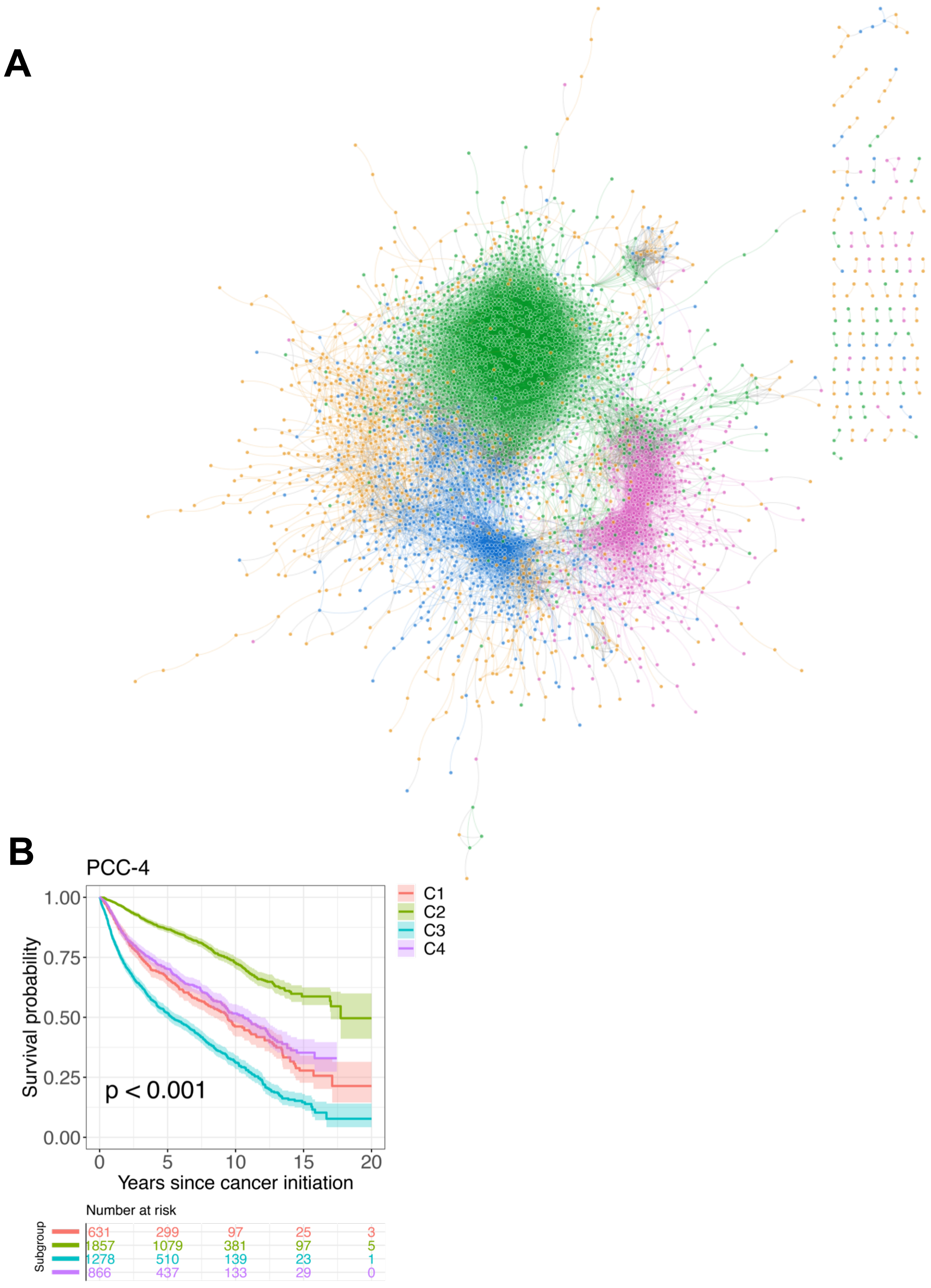

S4 Fig. Pearson correlation coefficient (PCC) as patient similarity metric. (A) Patient-patient network colorized by four cluster numbers. All edges have PCC < 0.65 for the patient pairs. All data preprocessing and PCC cutoff selection were same with the method cosine similarity calculation. The network was visualized using Cytoscape v 3.7.1. (**B)** Kaplan-Meier curves to estimate the all cause survival probability in the four subgroups. The Log-rank test were used to evaluate the statistical significance.
