# Supplementary material for: Cardiac risk stratification in cancer patients: A longitudinal patient–patient network analysis
Source: PLoS Med. 2021 Aug 2;18(8):e1003736. doi: 10.1371/journal.pmed.1003736 (PMC8366997; doi:10.1371/journal.pmed.1003736)
Supplement: S5 Fig — 5%, 10%, 15%, and 20% were used to test the K% connections with the highest PCC for the construction of the network. PCC, Pearson correlation coefficient. (PDF) [file pmed.1003736.s006.pdf]

S5 Fig

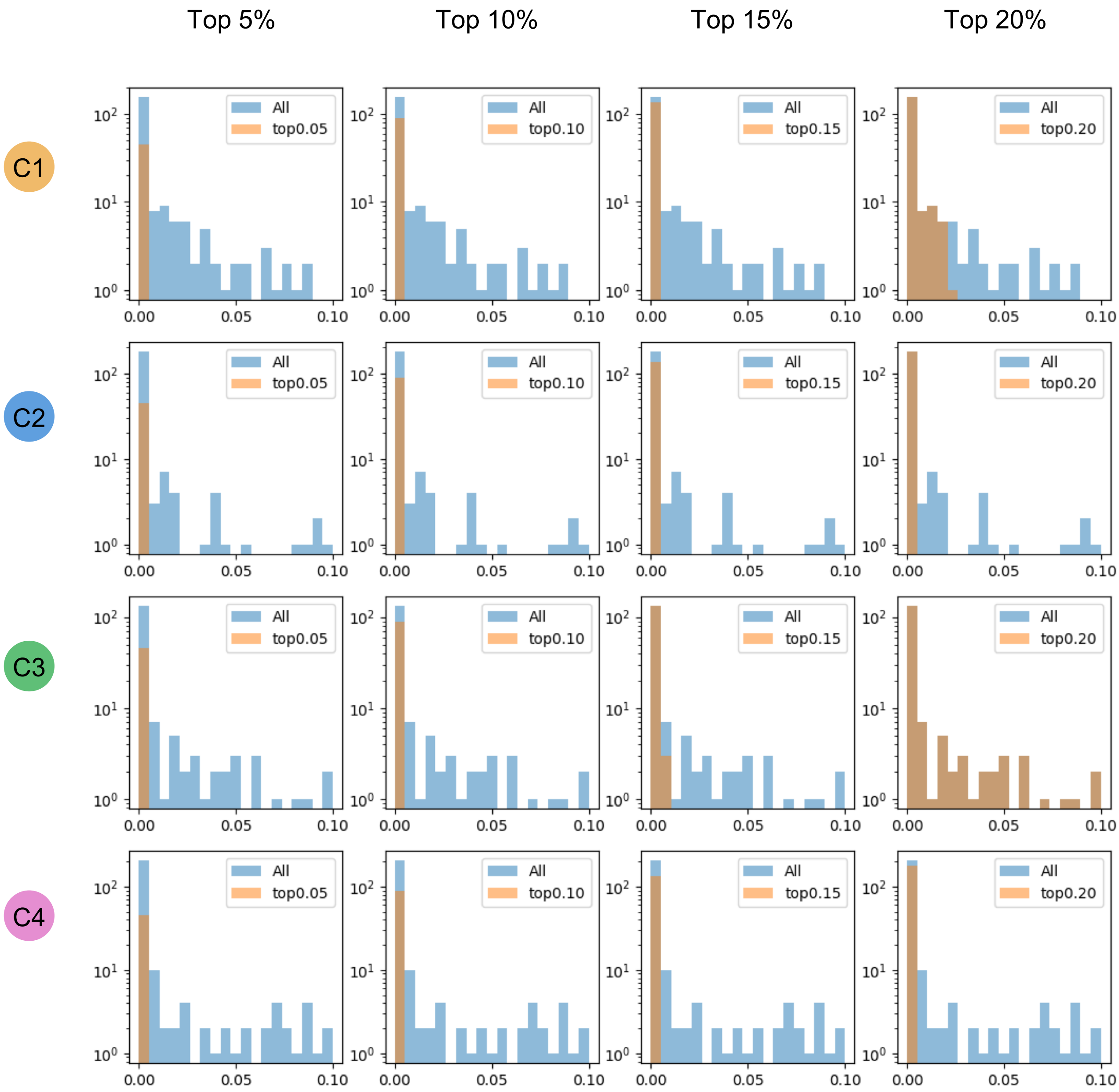

S5 Fig. Variable network PCC cutoff selection. 5%, 10%, 15%, and 20% were used to test the K% connections with the highest PCC for the construction of the network
