# Supplementary material for: Cardiac risk stratification in cancer patients: A longitudinal patient–patient network analysis
Source: PLoS Med. 2021 Aug 2;18(8):e1003736. doi: 10.1371/journal.pmed.1003736 (PMC8366997; doi:10.1371/journal.pmed.1003736)
Supplement: S6 Fig — (A) Cumulative hazard of 5 de novo CVD events across 4 subgroups are shown. The log-rank test with the BH adjustment was used for comparing the cumulative hazard among 4 subgroups. The shadow represents 95% CI. (B) The percentage of 5 CVD events across 4 subgroups. (C) The percentage of 5 de novo CVD events (the patient has at least one type of cardiac event diagnosed after cancer therapy) across 4 subgroups. AF, atrial fibrillation; BH, Benjamini and Hochberg; CAD, coronary artery disease; CI, confidence interval; CVD, cardiovascular disease; HF, heart failure; MI, myocardial infarction. (PDF) [file pmed.1003736.s007.pdf]

S6 Fig

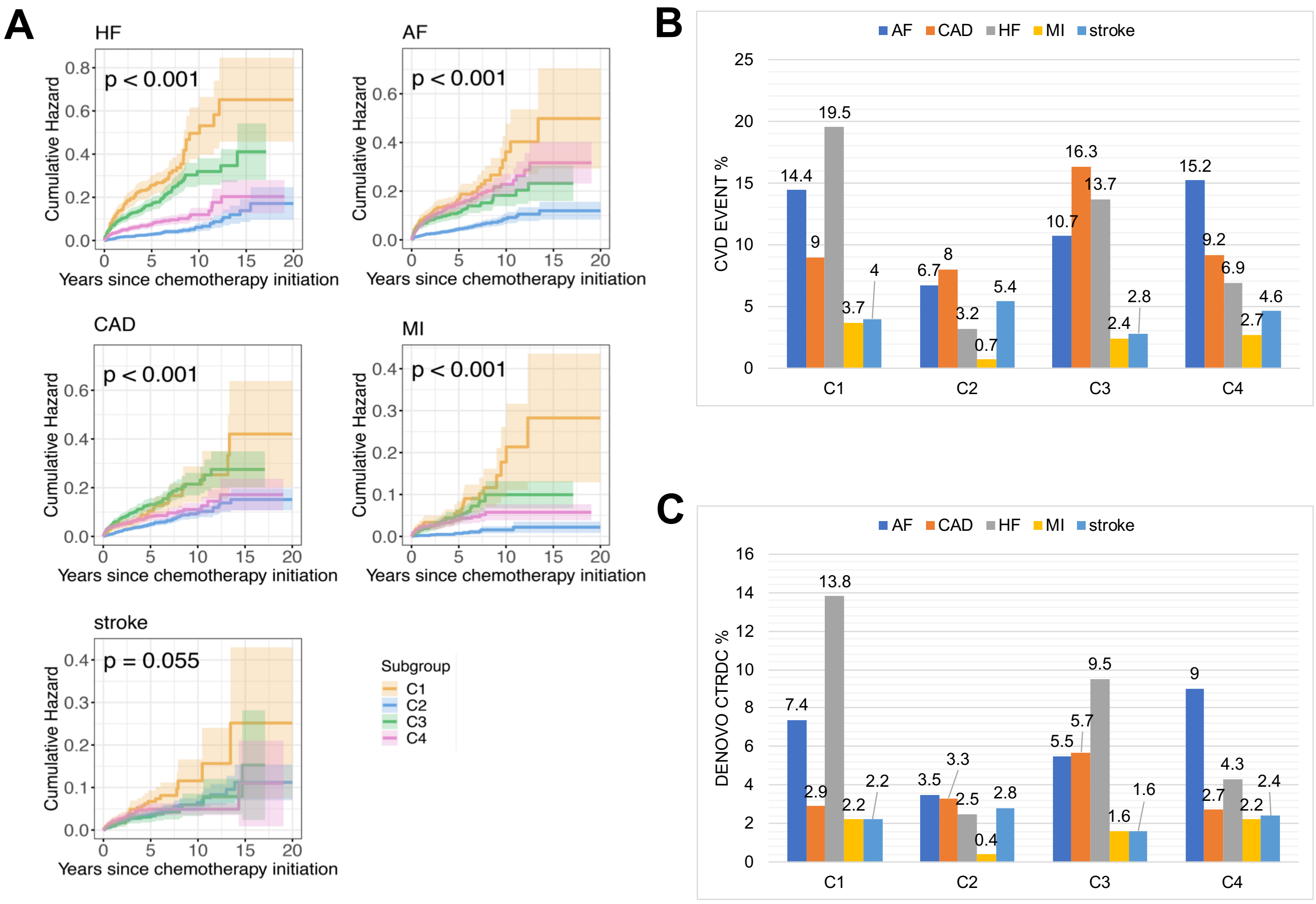

S6 Fig. Efficacy of the risk stratification on each cardiovascular disease (CVD) outcomes. (**A**) Cumulative hazard of 5 *de novo* CVD events across 4 subgroups are shown. The log-rank test with the Benjamini & Hochberg (BH) adjustment was used for comparing the cumulative hazard among 4 subgroups. The shadow represents 95% confidence interval. (**B**) The percentage of 5 CVD events across four subgroups. (**C**) The percentage of 5 *de novo* CVD events (the patient has at least one type of cardiac event diagnosed after cancer therapy) across four subgroups.
