# Supplementary material for: Cardiac risk stratification in cancer patients: A longitudinal patient–patient network analysis
Source: PLoS Med. 2021 Aug 2;18(8):e1003736. doi: 10.1371/journal.pmed.1003736 (PMC8366997; doi:10.1371/journal.pmed.1003736)
Supplement: S7 Fig — HRs (and 95% CI) of CTRCD, cancer type, and cancer stage aim to mortality outcome. The Wald χ2 test was used to evaluate the variables with statistically significant coefficients. CI, confidence interval; CTRCD, cancer therapy–related cardiac dysfunction; CVD, cardiovascular disease; HR, hazard ratio. (PDF) [file pmed.1003736.s008.pdf]

S7 Fig

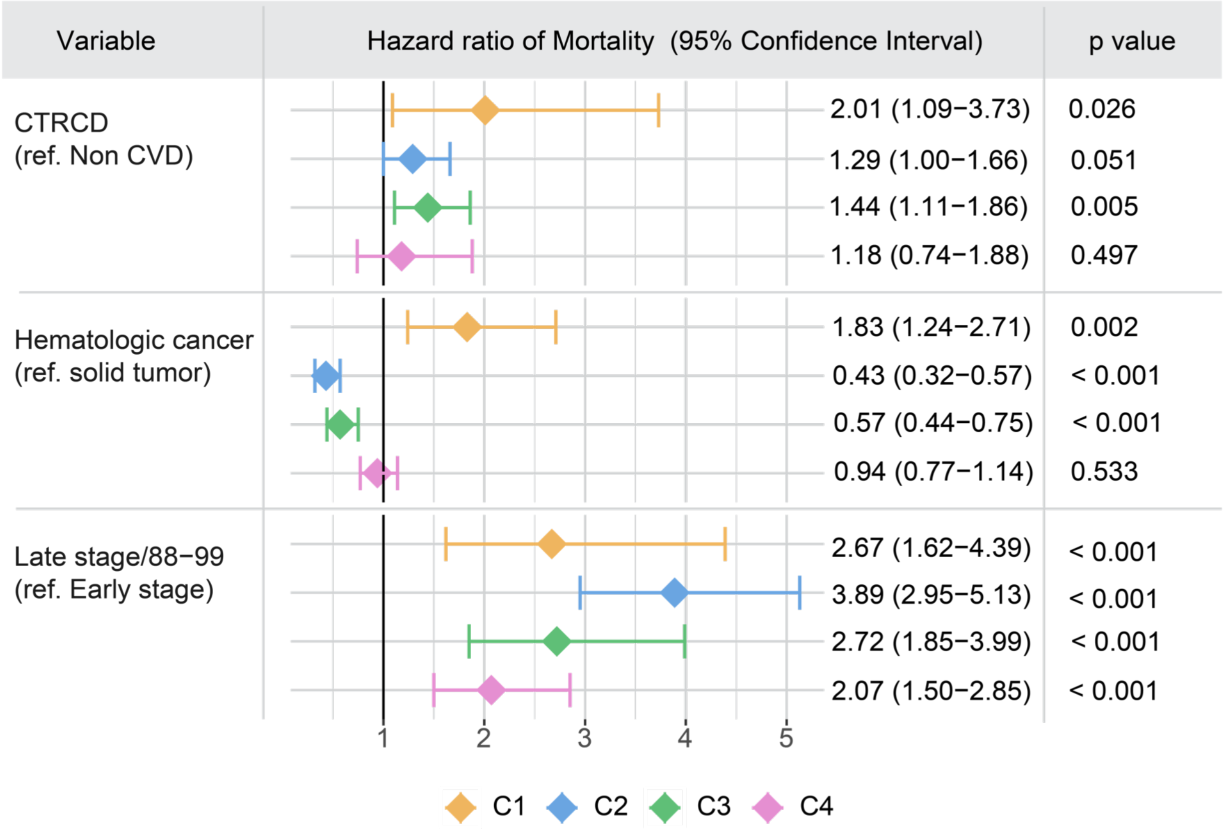

S7 Fig. Hazard ratio of mortality for four subgroups. Hazard ratios (and 95% confidence interval) of CTRCD, cancer type and cancer stage aim to mortality outcome. The Wald χ^2 test was used to evaluate the variables with statistically significant coefficients.
