# Supplementary material for: Cardiac risk stratification in cancer patients: A longitudinal patient–patient network analysis
Source: PLoS Med. 2021 Aug 2;18(8):e1003736. doi: 10.1371/journal.pmed.1003736 (PMC8366997; doi:10.1371/journal.pmed.1003736)
Supplement: S8 Fig — (A) KM curves to estimate all survival probability and (B) cumulative hazard of de novo CTRCD (the patient has at least one type of cardiac event diagnosed after cancer therapy) across 4 subgroups are shown. The log-rank test with the BH adjustment was used for comparing the cumulative hazard among 4 subgroups. The shadow represents 95% CI. BH, Benjamini and Hochberg; CI, confidence interval; CTRCD, cancer therapy–related cardiac dysfunction; KM, Kaplan–Meier. (PDF) [file pmed.1003736.s009.pdf]

S8 Fig

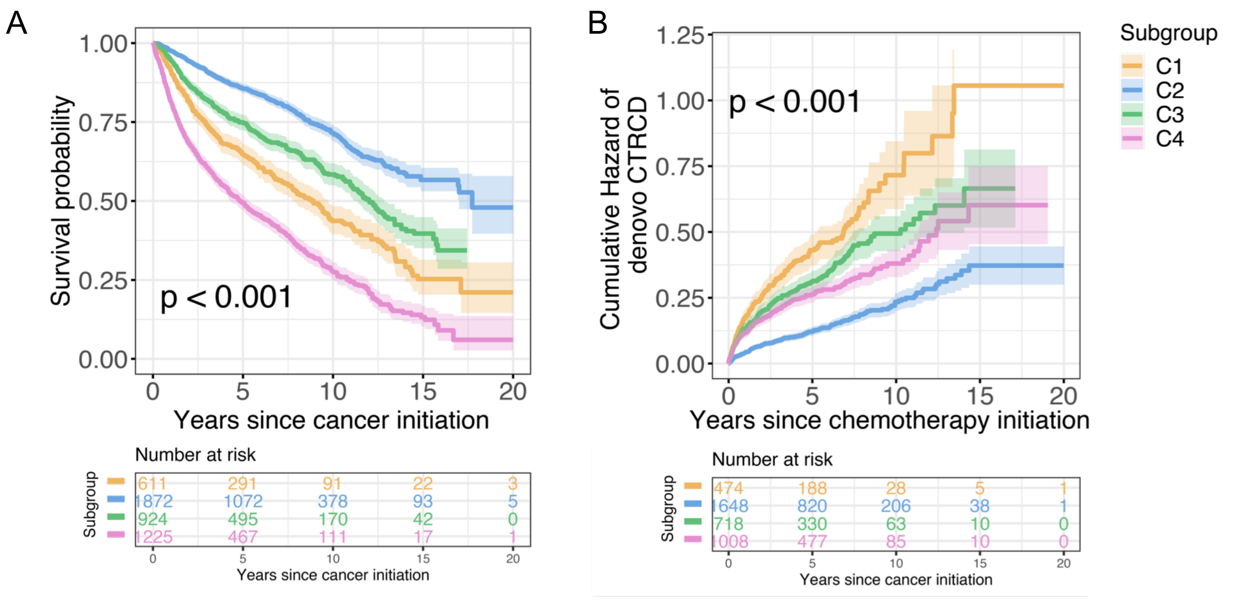

S8 Fig. Outcome validation for risk stratification model on the clinically-derived variables plus cancer type, cancer stage and treatment type. (**A**) Kaplan-Meier curves to estimate all survival probability and **(B)** Cumulative hazard of *de novo* CTRCD (the patient has at least one type of cardiac event diagnosed after cancer therapy) across 4 subgroups are shown. The log-rank test with the Benjamini & Hochberg (BH) adjustment was used for comparing the cumulative hazard among 4 subgroups. The shadow represents 95% confidence interval.
