# Supplementary material for: Cardiac risk stratification in cancer patients: A longitudinal patient–patient network analysis
Source: PLoS Med. 2021 Aug 2;18(8):e1003736. doi: 10.1371/journal.pmed.1003736 (PMC8366997; doi:10.1371/journal.pmed.1003736)
Supplement: S10 Fig — AF, atrial fibrillation; CAD, coronary artery disease; CTRCD, cancer therapy–related cardiac dysfunction; HF, heart failure; MI, myocardial infarction. (PDF) [file pmed.1003736.s011.pdf]

# S10 Fig

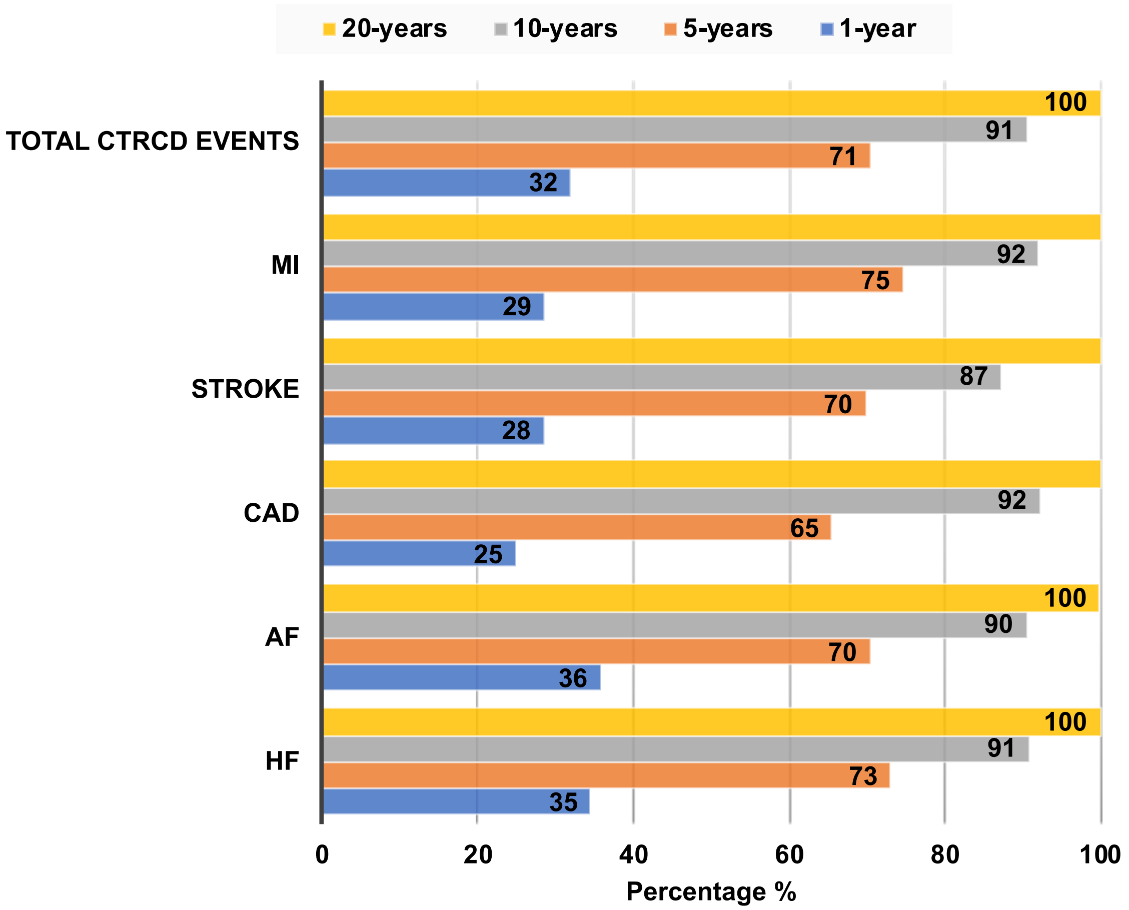


**S10 Fig. Cumulative percentage of five *de novo* CTRCD events from chemotherapy initiation one year, 5 years, 10 years and 20 years.**
