# Supplementary material for: Cardiac risk stratification in cancer patients: A longitudinal patient–patient network analysis
Source: PLoS Med. 2021 Aug 2;18(8):e1003736. doi: 10.1371/journal.pmed.1003736 (PMC8366997; doi:10.1371/journal.pmed.1003736)
Supplement: S11 Fig — (A) Betweenness centrality of clinical variables across 4 patient subgroup-specific clinical variable network. The gradient bar shows the centrality range. (B) Lab testing values for 4 selected clinical variables across different patient subgroups. The vertical bar denotes the 25% to 75% range, and the thick horizontal lines in each bean plot represent the average value. The black asterisk (*) denotes statistically significantly clinical variables in specific patient subgroup compared to the C2 subgroup. p-value was computed by KS test. All statistical data are provided in S8 Table. BSA, body surface area; ESV, end-systolic volume; KS, Kolmogorov–Smirnov. (PDF) [file pmed.1003736.s012.pdf]

S11 Fig

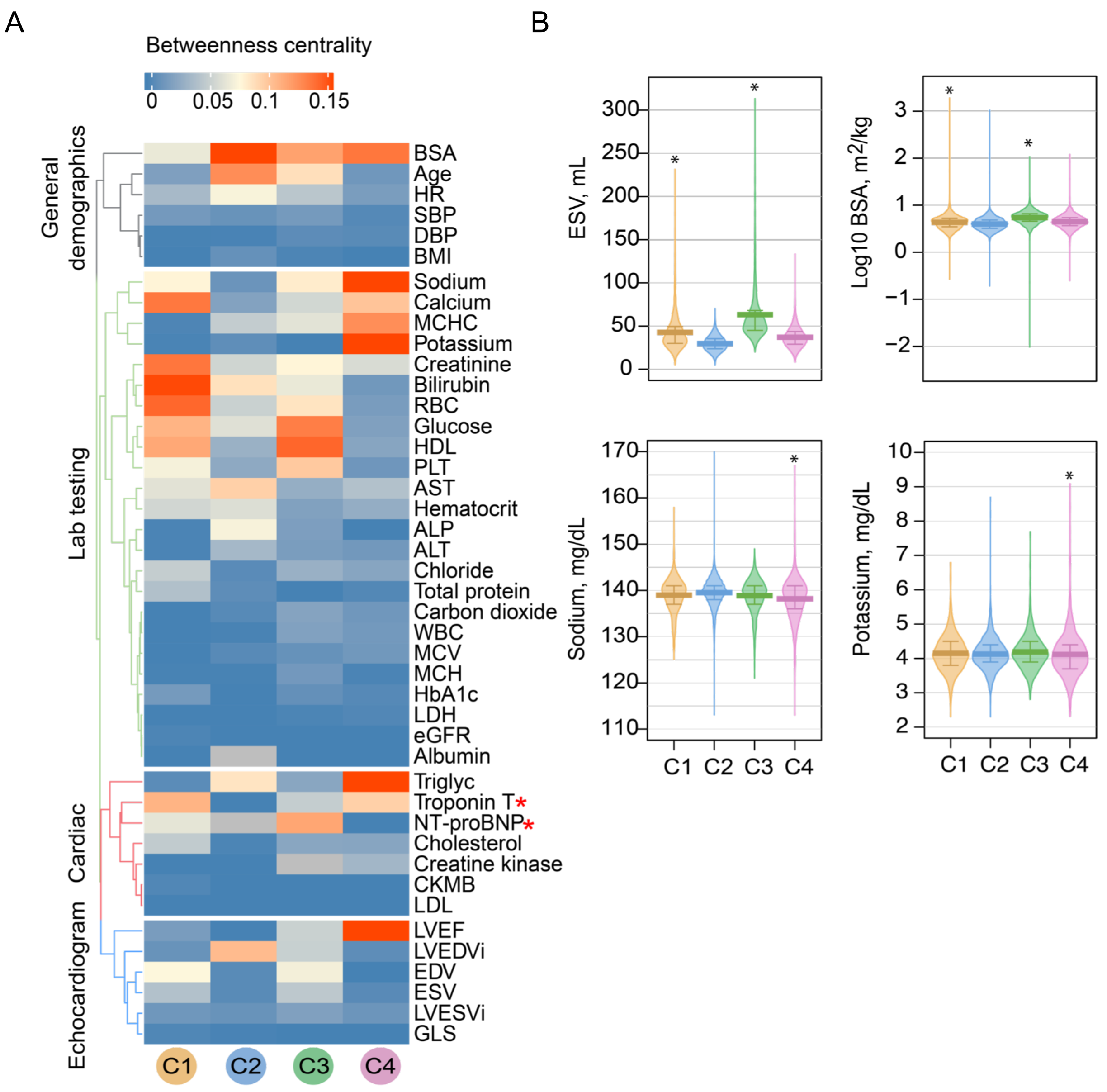

S11 Fig. Betweenness centrality of the variables. **(A)** Betweenness centrality of clinical variables across four patient subgroup-specific clinical variable network. The gradient bar shows the centrality range. (**B)** Lab testing values for four selected clinical variables across different patient subgroups. The vertical bar denotes the 25% to 75% range and the thick horizontal line in each bean plot represent the average value. ESV: end-systolic volume, BSA: body surface area. The black asterisk (*) denotes statistically significantly clinical variables in specific patient subgroup compared to the C2 subgroup. P-value was computed by Kolmogorov–Smirnov test. All statistical data are provided in supplementary Table S1.
