# Supplementary material for: Cardiac risk stratification in cancer patients: A longitudinal patient–patient network analysis
Source: PLoS Med. 2021 Aug 2;18(8):e1003736. doi: 10.1371/journal.pmed.1003736 (PMC8366997; doi:10.1371/journal.pmed.1003736)
Supplement: S12 Fig — The gradient red color denotes positive correlation, and gradient blue color denotes negative correlation. The order of labels in heatmap were followed by 4 variable categories. Due to the space limitation, the labels in heatmap show one name in every 3 names. The full correlation matrix of 112 variables were showed in S10 Table, and the order of variable labels were the same with the label ranked in the heatmap. (PDF) [file pmed.1003736.s013.pdf]

# S12 Fig

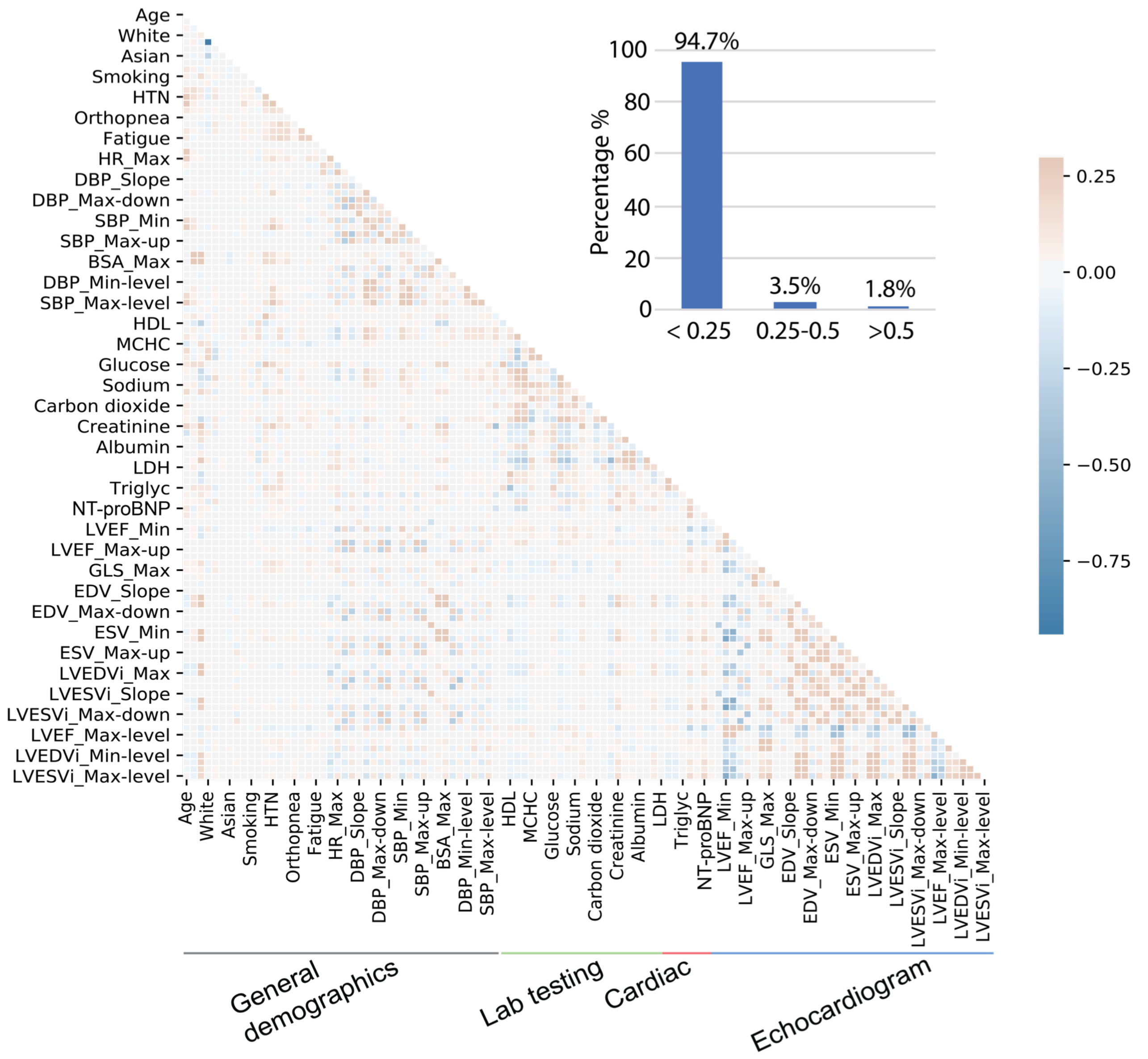


**S12 Fig. Pairwise Pearson Correlations among the used 112 clinical variables.** The gradient red color denotes positive correlation and gradient blue color denotes negative correlation. The order of labels in heatmap were followed by four variable categories. Duo to the space limitation, the labels in heatmap show one name in every 3 names. The full correlation matrix of 112 variables were showed in S10 Table, and the order of variable labels were the same with the label ranked in the heatmap.
