# Supplementary material for: Cardiac risk stratification in cancer patients: A longitudinal patient–patient network analysis
Source: PLoS Med. 2021 Aug 2;18(8):e1003736. doi: 10.1371/journal.pmed.1003736 (PMC8366997; doi:10.1371/journal.pmed.1003736)
Supplement: S13 Fig — All patients were split randomly or by time to training and test sets. We computed the cosine similarity matrix for patients in the training set (blue matrix) and for patients in the test set (green matrix) against the training set. Next, the K-means clustering was performed on the training set and was used to predict both the training and test sets. The predicted clusters were evaluated for the survival and de novo CTRCD risk for both the training and test sets. CTRCD, cancer therapy–related cardiac dysfunction; psnCVD, patient–patient similarity network-based risk assessment of CVD. (PDF) [file pmed.1003736.s014.pdf]

S13 Fig

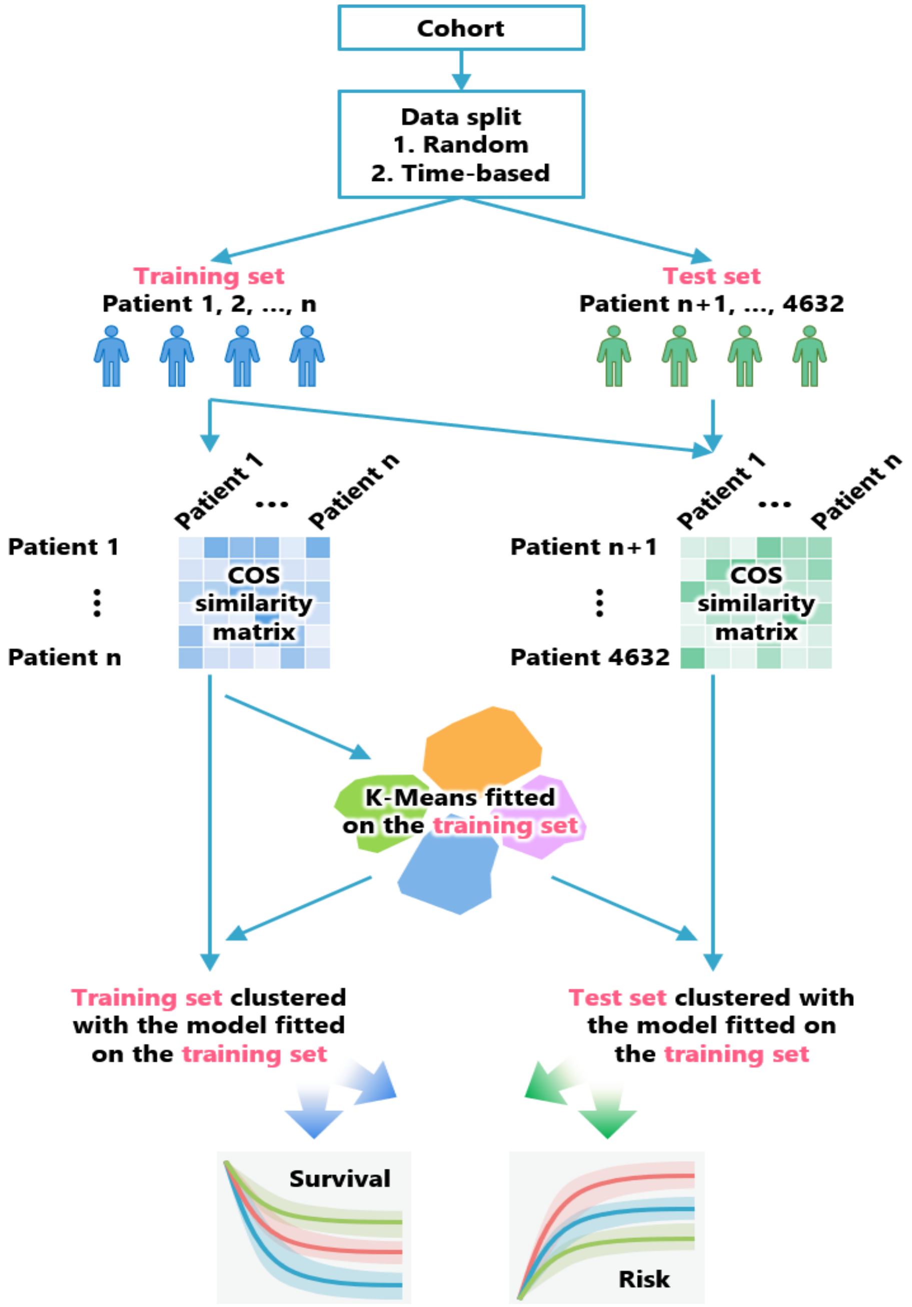

S13 Fig. The workflow of the train-test validation strategy to test the generalizability of the clustering models. All patients were split randomly or by time to training and test sets. We computed the cosine similarity matrix for the training patients (blue matrix) and for the test patients (green matrix) against the training patients. Next, the K-means clustering was performed on the training set and was used to predict both the training and test sets. The predicted clusters were evaluated for the survival and de novo CTRCD risk for both the training and test sets.
