# Supplementary material for: Cardiac risk stratification in cancer patients: A longitudinal patient–patient network analysis
Source: PLoS Med. 2021 Aug 2;18(8):e1003736. doi: 10.1371/journal.pmed.1003736 (PMC8366997; doi:10.1371/journal.pmed.1003736)
Supplement: S14 Fig — Patients were split by their cancer diagnosis time to 3 training set/test set pairs: 50% versus 50%, 60% versus 40%, and 80% versus 20%, respectively. The survival probability and cumulative hazard of de novo CTRCD of the training sets and test sets were evaluated. Log-rank tests show statistically significant difference in survival probability and cumulative hazard of de novo CTRCD for the patient groups in the test sets. CTRCD, cancer therapy–related cardiac dysfunction; psnCVD, patient–patient similarity network-based risk assessment of CVD. (PDF) [file pmed.1003736.s015.pdf]

S14 Fig

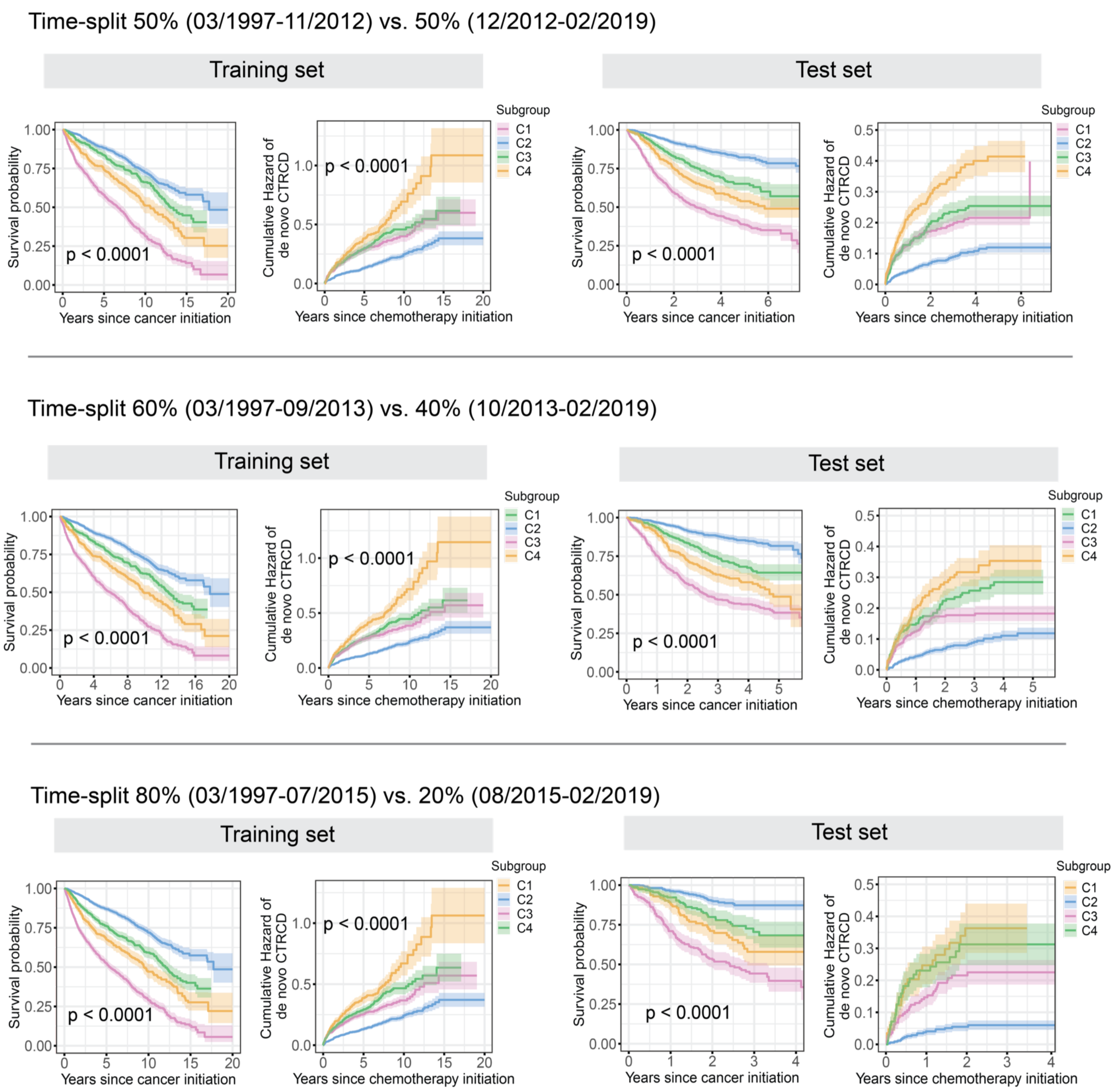

S14 Fig. Evaluation of the generalizability of network-based K-means clustering using time split cohorts. Patients were split by their cancer diagnosis time to three training set / test set pairs that were 50% versus 50%, 60% versus 40%, and 80% versus 20%, respectively. The survival probability and cumulative hazard of *de novo* CTRCD of the training sets and test sets were evaluated. Log-rank tests show statistically significant difference in survival probability and cumulative hazard of de novo CTRCD for the patient groups in the test sets, consistent with that of the training sets.
