# Supplementary material for: Cardiac risk stratification in cancer patients: A longitudinal patient–patient network analysis
Source: PLoS Med. 2021 Aug 2;18(8):e1003736. doi: 10.1371/journal.pmed.1003736 (PMC8366997; doi:10.1371/journal.pmed.1003736)
Supplement: S15 Fig — The survival probability and cumulative hazard of de novo CTRCD of the training set (50%) and test set (50%) were evaluated in 3 independent random experiments. Log-rank tests show statistically significant difference in survival probability and cumulative hazard of de novo CTRCD for the patient groups in the test sets. CTRCD, cancer therapy–related cardiac dysfunction; psnCVD, patient–patient similarity network-based risk assessment of CVD. (PDF) [file pmed.1003736.s016.pdf]

S15 Fig

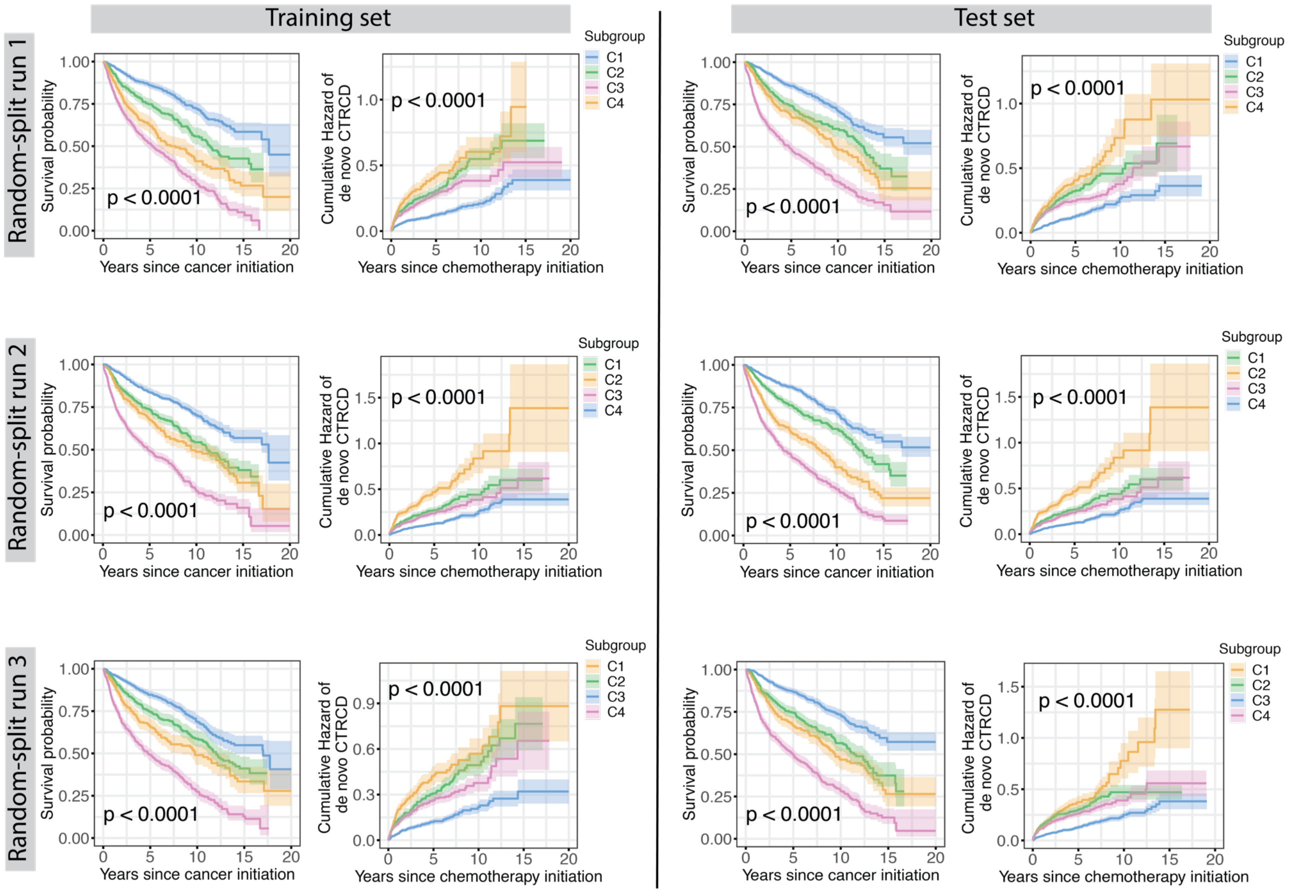

S15 Fig. Evaluation of the generalizability of network-based K-means clustering using random split cohorts. The survival probability and cumulative hazard of de novo CTRCD of the training set (50%) and test set (50%) were evaluated in three independent random experiments. Log-rank tests show statistically significant difference in survival probability and cumulative hazard of de novo CTRCD for the patient groups in the test sets, consistent with that of the training sets.
