# Supplementary material for: Cardiac risk stratification in cancer patients: A longitudinal patient–patient network analysis
Source: PLoS Med. 2021 Aug 2;18(8):e1003736. doi: 10.1371/journal.pmed.1003736 (PMC8366997; doi:10.1371/journal.pmed.1003736)
Supplement: S16 Fig — Time-dependent AUROC analysis of Cox proportional hazard models using the entire cohort (A) and individual patient subgroups identified by psnCVD models (B–E). The overall performance of Cox proportional hazards model using the entire cohort (A) and individual patient subgroups (B–E). For each subplot, all patients (A) or patients in individual subgroups (B–E) were randomly split to training (50%) and test (50%) set. The clusters for the patients in the test set were predicted based on the model fitted on the training set. Time-dependent AUROC was used to evaluate the model performance of the test sets. AUROC, area under the receiver operating characteristic curve; psnCVD, patient–patient similarity network-based risk assessment of CVD. (PDF) [file pmed.1003736.s017.pdf]

S16 Fig

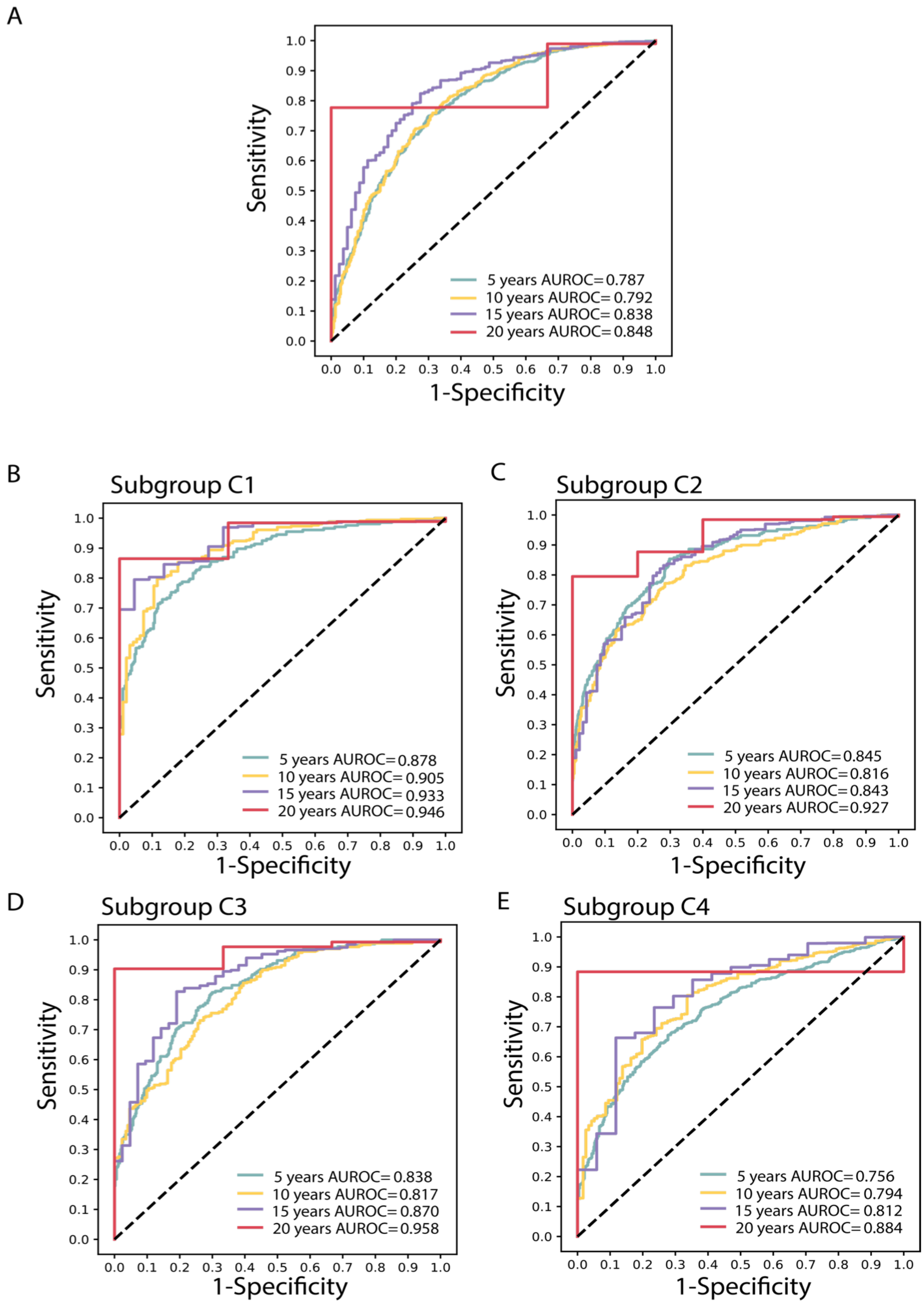

S16 Fig. Time-dependent AUROC analysis of Cox proportional hazard models using the entire cohort (A) and individual patient subgroups identified by psnCVD models (B-E). The overall performance of Cox proportional hazards model using the entire cohort (**A**) and individual patient subgroups (**B-E**). For each subplot, all patients (A) or patients in individual subgroups (**B-E**) were randomly split to training (50%) and test (50%) set. The clusters for the patients in the test set were predicted based on the model fitted on the training set. Time-dependent AUROC was used to evaluate the model performance of the test sets**.**
