# Supplementary material for: Cardiac risk stratification in cancer patients: A longitudinal patient–patient network analysis
Source: PLoS Med. 2021 Aug 2;18(8):e1003736. doi: 10.1371/journal.pmed.1003736 (PMC8366997; doi:10.1371/journal.pmed.1003736)
Supplement: S17 Fig — (A) Patient–patient network colorized by 3 cluster numbers. Patient–patient network using a subpopulation of patients (n = 1,252) who received chemotherapy only. Using cosine < 0.55 as a cutoff, 3 clusters were identified: cluster 1a (n = 502), cluster 2a (n = 474), and cluster 3a (n = 275). The network was visualized using Cytoscape v3.7.1. (B) Cumulative hazard of de novo CTRCD in the 3 subgroups. The log-rank test was used to evaluate the statistical significance. (C) KM curves to estimate the all-cause survival probability in the 3 subgroups. CTRCD, cancer therapy–related cardiac dysfunction; KM, Kaplan–Meier. (PDF) [file pmed.1003736.s018.pdf]

S17 Fig

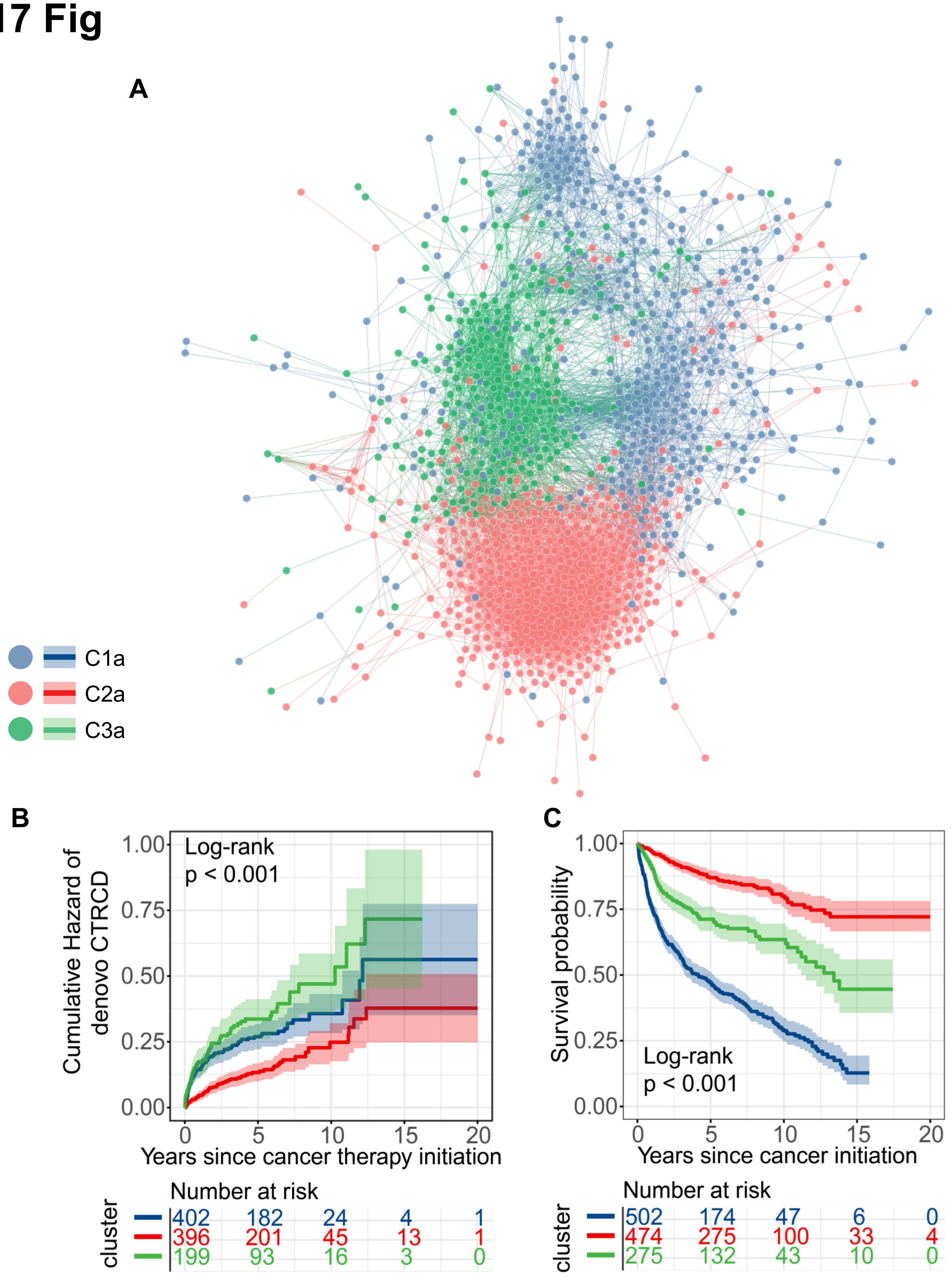

S17 Fig Methodology application in chemotherapy population

(**A**) Patient-patient network colorized by three cluster numbers. Patient-patient network using a sub-population of patients (n=1,252) who received chemotherapy only. Using cosine < 0.55 as a cutoff, 3 clusters were identified: cluster 1a (n=502), cluster 2a (n=474), and cluster 3a (n=275). The network was visualized using Cytoscape v 3.7.1. (**B**) Cumulative hazard of *de novo* CTRCD in the three subgroups. The Log-rank test were used to evaluate the statistical significance. (**C**) Kaplan-Meier curves to estimate the all cause survival probability in the three subgroups.
