# Supplementary material for: Cardiac risk stratification in cancer patients: A longitudinal patient–patient network analysis
Source: PLoS Med. 2021 Aug 2;18(8):e1003736. doi: 10.1371/journal.pmed.1003736 (PMC8366997; doi:10.1371/journal.pmed.1003736)
Supplement: Attachment — Submitted filename: Rebuttal_Letter_Dr.Cheng.pdf [file pmed.1003736.s030.pdf]

Manuscript #: PMEDICINE-D-20-00473R1

Dear Dr. Veitch and Reviewers

Thank you for your time and thoughtful feedback. The corresponding authors and other authors, have crafted the itemized response to your comments as below.

Responses to Reviewer #1

1. "Longitudinal Population-based Cardiac Risk Stratification in 4,600 Cancer Patients from 1997 to 2019" introduces a network clustering approach on longitudinal clinically-derived variables, to identify four clinically-relevant subgroups with correlations to cancer therapy-related cardiac dysfunction (CTRCD) outcomes. Analysis of the network further identified particular cardiac variables that may be actionable biomarkers associated with CTRCD. The use of clustering methods in grouping patients has been popular in medicine, likely due to the natural intuition that if two patients have similar profiles as far as is known, their outcomes should also be similar, or at least more than between patients with dissimilar profiles. The availability of the relevant code (with accompanying commentary) on GitHub was appreciated for aiding understanding.

Response: We thank the Reviewer for the great summary and the overall positive feedback regarding our study.

2. However, there remain two relatively major concerns that might be addressed: Firstly, for this manuscript, a patient-patient similarity network was constructed before (K-means) clustering was employed. The cosine similarity metric (Equation 1; also commonly seen in evaluating embedding distances) was employed to determine whether two patients are sufficiently-similar (i.e. have cosine similarity above some cutoff), and thus connected in the similarity network. While not explicitly stated, it is implied from S1 Fig that if a particular patient has no similar-enough fellows, that patient is not included in the similarity network (because as the cutoff threshold increases, the number of patients/nodes in the network decreases with increasing number of disconnected nodes).

Response: We thank the Reviewer for this good point regarding the rationale of the study. We have added more detailed methodologies for network construction and visualization in the revised method. To be specific, we used total 4,632 patients for K-means clustering to generate our final patient-patient network. Thus, patients having high and low similarities are included for K-means clustering. Subsequently, we performed the survival outcome analysis to validate the final clustering results for 3,131 patients as patients without survival data are excluded (**Fig 2C**). To respond the Reviewer's concerns, we performed new K-means clustering analysis using the raw clinical variables of total 4,632 patients. We found that K-means clustering on the raw clinical variables cannot identify four clusters validated by survival outcomes and cardiovascular events in the **extended Figure 1** (please see more detailed responses to the Comment #6).

In summary, we included all 4,632 patients for K-means clustering and the cutoff threshold we used to minimize network density dose not influence of performance of our proposed patient-patient network models.

3. 1a. It is not explicitly explained why a similarity network with minimized network density (i.e. as few links as possible, for a given maximum possible number of links as determined by the number of nodes in the network) is desirable. Intuitively, links between nodes in a network with low density might be considered more "meaningful", since they are relatively rare. However, the tradeoff (as seen in S1 Fig) is that a proportion of patients would have no links due to the cutoff threshold, and will thus be excluded from all subgroups (from Page 10, the final network contains only 3,131 of the original 4,632 patients). The authors might consider more formally explaining the choice of minimized network density, with references if appropriate.

Response: We thank the Reviewer for confirming that links between nodes in a network with low density should be considered more "meaningful" in the real-world networks. This is the network theory (Lawyer, *Scientific Reports* 2015) we used to identify a minimized network density for "meaningful" findings.

In this study, we use the cutoff threshold to minimize network density of patient-patient networks based on previous studies (Feng et al., *Chemistry & Biodiversity* 2012,

Mao et al., *BMC Bioinformatics* 2009), which help us balance the network size and clinically "meaningful" discoveries. This strategy has been widely adopted by several previous patient-patient network studies (Li et al., *Science Translational Medicine* 2015 and Oldham et al., *Circulation Research* 2018).

In this study, we used all 4,632 patients for K-means clustering and we only selected 3,131 patients to visualize the patient-patient network in **Figure 2A** using a specific threshold by minimizing network density. It will be a hairball network if we visualized full patient similarity matrices of 4,632 x 4,632. To respond the Reviewer's concern, we have added more explanation about how we minimize network density in the revised manuscript.

References:

1. Lawyer, G. (2015). Understanding the influence of all nodes in a network. *Scientific Reports* 5, 8665.
2. Feng, Y., Hurst, J., Almeida-De-Macedo, M., Chen, X., Li, L., Ransom, N., and Wurtele, E.S. (2012). Massive human co-expression network and its medical applications. *Chemistry & Biodiversity* 9, 868-887.
3. Mao, L., Van Hemert, J.L., Dash, S., and Dickerson, J.A. (2009). Arabidopsis gene co-expression network and its functional modules. *BMC Bioinforma*tics 10, 346.
4. Li, L., Cheng, W.-Y., Glicksberg, B.S., Gottesman, O., Tamler, R., Chen, R., Bottinger, E.P., and Dudley, J.T. (2015). Identification of type 2 diabetes subgroups through topological analysis of patient similarity. *Science Translational Medicine* 7, 311ra174-311ra174.
5. Oldham, W.M., Oliveira, R.K.F., Wang, R.S., Opotowsky, A.R., Rubins, D.M., Hainer, J., Wertheim, B.M., Alba, G.A., Choudhary, G., Tornyos, A., et al. (2018). Network analysis to risk stratify patients with exercise intolerance. *Circulation research* 122, 864-876.

4. Further on minimizing network density, there appears to be no guarantee that a cutoff that yields minimum density would also retain a meaningful number of patients/nodes

(e.g. from S1 Fig, fewer than 1000 patients/nodes remain at a cutoff of 0.75). Does this possibility factor into the choice of the network density metric?

Response: Yes, this possibility factor was also considered when choosing the cutoff. The chosen cutoff was based on a compromise between retaining more patients for the network visualization and obtaining a lower network density. We have discussed this limitation in the revised manuscript.

5. 1b. It is then stated that patients were clustered "using their network profiles". "Network profile" does not seem to be defined in the text, but from the GitHub, it appears that a patient's profile consists of all other similar patients (by cosine similarity), but without the similarity value (i.e. once above the cutoff, it is not considered whether the patients are barely sufficiently similar, or essentially identical). The definition of network profile might be provided in the text.

Response: As stated in the original GitHub document, we used "the entire of the cosine similarity matrix including all 4,632 x 4,632 patient-patient similarities" as the network profile. We have added more detailed explanation for network construction and visualization in the revised Methods section. We updated the description in GitHub as "To identify patient subgroups, we clustered the patients using their network profiles (the entire cosine similarity matrix including all patient-patient similarities) by K-means clustering analysis."

6. 1c. Following from 1a & 1b, it is unclear why the similarity network is necessary. The K-means clustering could conceivably have been directly performed on the clinically-derived variables for all patients, as would seem the usual practice. This would moreover not detract from interpretability, since all patients would remain as nodes in a topological space. The authors might cite/describe theoretical support for psnCVD, and/or empirical evidence that it is superior to direct K-means clustering as a baseline.

Response: We thank the Reviewer for this critique. We have performed K-means clustering directly on the raw clinical variables for all 4,632 patients. We found that K-means clustering from raw clinical variables cannot separate two cardiac-risk subgroups (the *orange* subgroup and *green* subgroup, **Extended Figure 1** referring to revised **S6**

Fig), comparing to K-means clustering from the patient-patient similarity network (**Fig 2B**). In this study, we presented psnCVD based on several recent patient network theoretical studies (Li et al., *Science Translational Medicine* 2015; Oldham et al., *Circulation Research* 2018; Pai et al., *Molecular Systems Biology* 2019). We have added more explanations in page 16 of the revised manuscript.

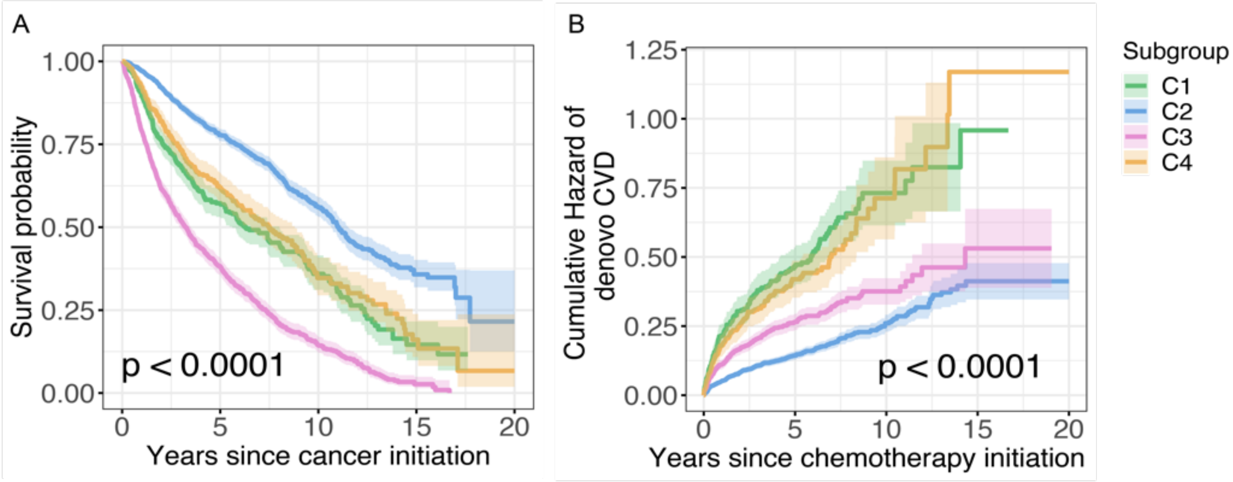

Extended Figure 1. Outcome validation for K-means clustering directly on the raw clinical variables for all 4,632 patients. (**A**) Kaplan-Meier curves to estimate all survival probability across 4 subgroups are shown and (**B**) Cumulative hazard of *de novo* CTRCD (the patient has at least one type of cardiac event diagnosed after cancer therapy). The log-rank test with the Benjamini & Hochberg (BH) adjustment was used for comparing the cumulative hazard among 4 subgroups. The shadow represents 95% confidence interval.

References:

1. Li, L., Cheng, W.-Y., Glicksberg, B.S., Gottesman, O., Tamler, R., Chen, R., Bottinger, E.P., and Dudley, J.T. (2015). Identification of type 2 diabetes subgroups through topological analysis of patient similarity. *Science Translational Medicine* 7, 311ra174-311ra174.
2. Oldham, W.M., Oliveira, R.K.F., Wang, R.S., Opotowsky, A.R., Rubins, D.M., Hainer, J., Wertheim, B.M., Alba, G.A., Choudhary, G., Tornyos, A., et al. (2018). Network analysis to risk stratify patients with exercise intolerance. *Circulation research* 122, 864-876.

3. Pai, S., Hui, S., Isserlin, R., Shah, M.A., Kaka, H., and Bader, G.D. (2019). netDx: interpretable patient classification using integrated patient similarity networks. *Molecular Systems Biology* 15, e8497.

7. 1d. The x-axes for S1 Fig read "PCC cutoff", but Pearson correlation coefficient (PCC) appears to be an alternative metric to cosine similarity, which is supposed to be used in S1 Fig. Is this a mislabeling?

Response: We apologized for this mislabeling. We have changed "PCC cutoff" to "Cosine cutoff" in the revised **S2 Fig** (original **S1 Fig**).

8. 1e. On Page 10, it is stated that "we tested the cutoffs in an increment of 0.5". Might this be "...in increments of 0.05" instead?

Response: We have changed as "Using this method, we tested the cutoffs in an increment of 0.05." in page 11 of the revised manuscript.

9. 1f. Given that a cutoff of 0.65 was identified as resulting in minimum network density, why was the connection cutoff between patients then states ad (> 0.62) in the next sentence, rather than 0.65?

Response: To better visualize patient-patient networks, we computed the network density at different cutoff thresholds and selected the cutoff that resulted in the lowest network density. Using this method, we tested the cutoffs in an increment of 0.05, and identified that the lowest network density (0.24%, **S2 Fig**) was achieved when the cutoff was 0.65. However, at this cutoff, only 2,669 patients were retained. Therefore, the final cutoff was based on a compromise between retaining more patients for the network visualization and obtaining a lower network density. Finally, all patient pairs with cosine > 0.62 were considered connected in the network. We have added more explanation in the revised Methods and added these details in the shared Github document to improve the reproducibility of our methodologies.

10. Secondly, there may be concerns with the replicability of the findings:

2a. K-means clustering remains a machine learning technique that possibly overfits given data. As such, to ensure that clinical subgroups discovered via clustering are reliable, common practice is to reproduce the clustering on independent cohorts (e.g. in "Novel subgroups of adult-onset diabetes and their association with outcomes: a data-driven cluster analysis of six variables", Ahlqvist et al., The Lancet Diabetes & Endocrinology, 2018), or on held-out validation data.

Response: We thank the Reviewer for this good point. We agree with the Reviewer that it will be great we can validate our findings using an independent cohort. To the best knowledge of the authors, our Cardio-Oncology cohort is the largest cohort with up to 20 years' longitudinal patient data. We reached a few colleagues across the top U.S. medical centers, but we cannot find any similar cohorts with ours as independent validation cohort.

To respond the Reviewer's concerns, we performed new patient-patient network analysis using two internal validation approaches: 1) three new subgroups split by patient follow-up time windows (**Extended Figure 2** referring to revised **S12 Fig**); and 2) a random sampling cohort with 50% patients (**Extended Figure 3** referring to revised **S13 Fig**). As shown in **extended Figures 2** and **3**, we re-identified four similar patient clusters which were well validated by survival and cardiovascular outcomes as well. These new internal validations reveal high reproducibility of our network-based methodologies. We agreed that we should validate it further using independent cohorts in our future studies. We have added more explanations and cited the abovementioned paper (Ahlqvist et al. 2018) in the revised manuscript.

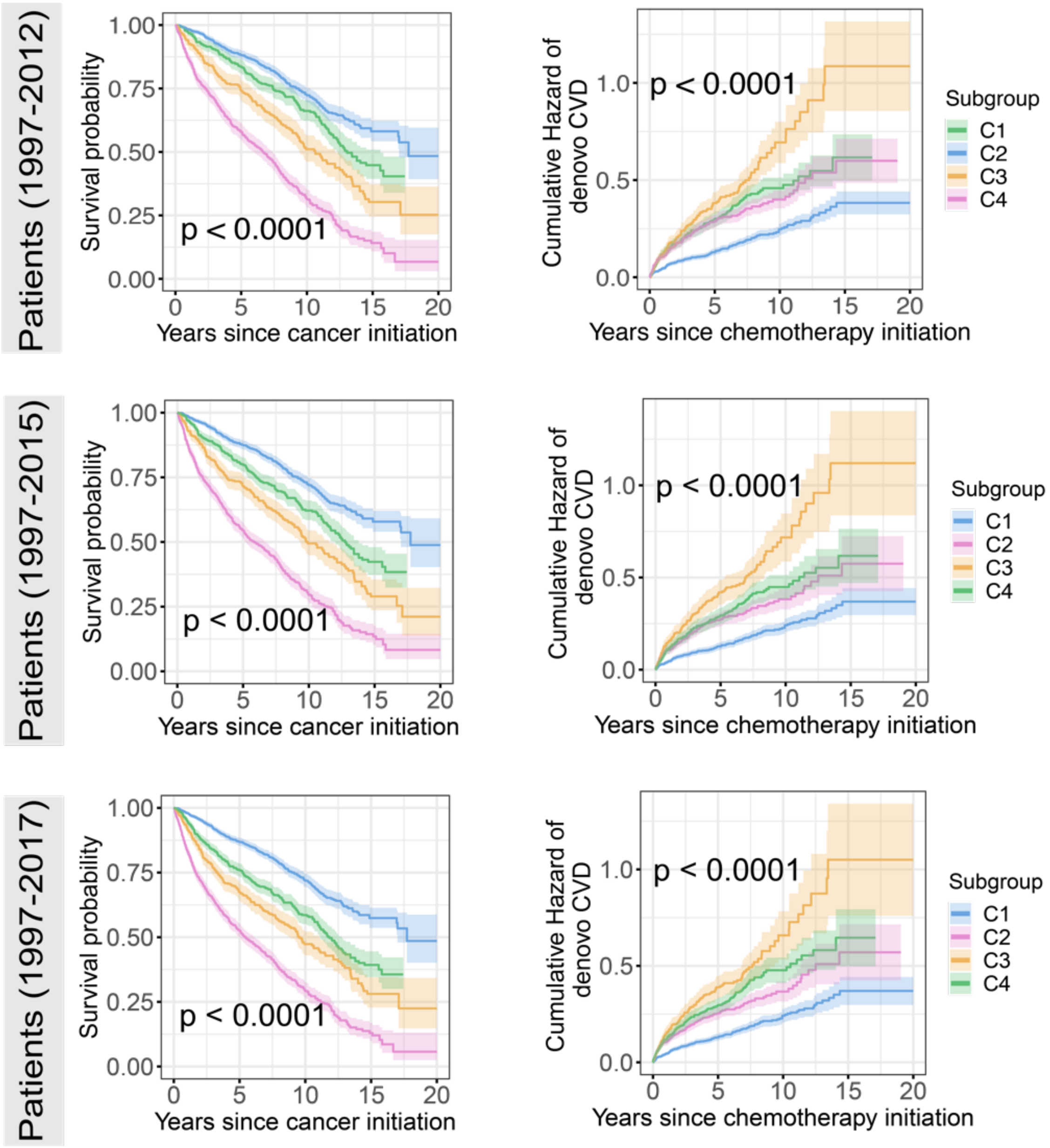

Extended Figure 2. Performance of network-based K-means clustering in three new subgroup (split by patient's follow-up time windows) analyses. The survival probability and cumulative hazard of *de novo* CTRCD across 4 patient clusters for three new subgroups analyses: i) Patients (1997-2012): a sub-cohort includes patients who were diagnosed cancer during 1997 to 2012; ii) Patients (1997-2015): a sub-cohort includes patients who were diagnosed cancer during 1997 to 2015; and iii) Patients (1997-2017): a sub-cohort includes patients who were diagnosed cancer during 1997 to 2017.

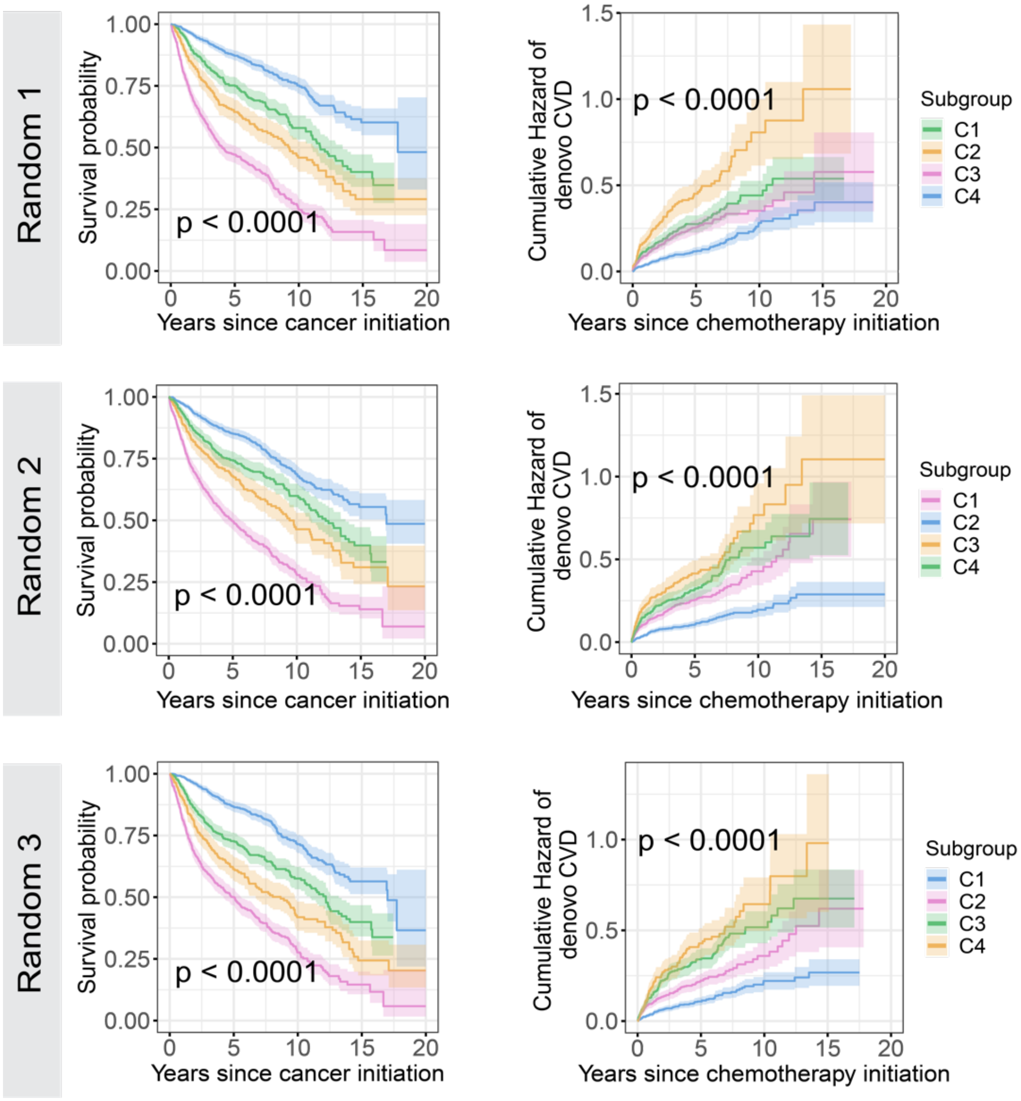

Extended Figure 3. Performance of network-based K-means clustering in random sampling cohorts. The survival probability and cumulative hazard of *de novo* CTRCD across 4 new network-predicted clusters in a random sampling cohort with three independent experiments. The sampling ratio is 50%.

11. 2b. K-means clustering may moreover have a stochastic component, resulting in somewhat different clusters being produced from the same input data. If so, the clusterwise stability might be assessed (also as in Ahlqvist et al.)

Response: We thank the Reviewer for this good point. We computed adjusted rand index (ARI) and adjusted mutual Information (AMI) to validate the clustering stability. For both metrics, a value of 1 indicates perfect agreement, while randomly assigned

clusters have scores around 0 (Hubert et al, *Journal of Classification* 1985; Vinh et al, *Journal of Machine Learning Research* 2010). Following the workflow (**Extended Figure 4** referring to revised **S1A Fig**), we performed 100 repeats K-means clustering, 99 repeats showed high ARI and AMI score for the clusters, indicating high stability of our proposed network-based K-means clustering methodology (**Extended Figure 4** referring to revised **S1B Fig**).

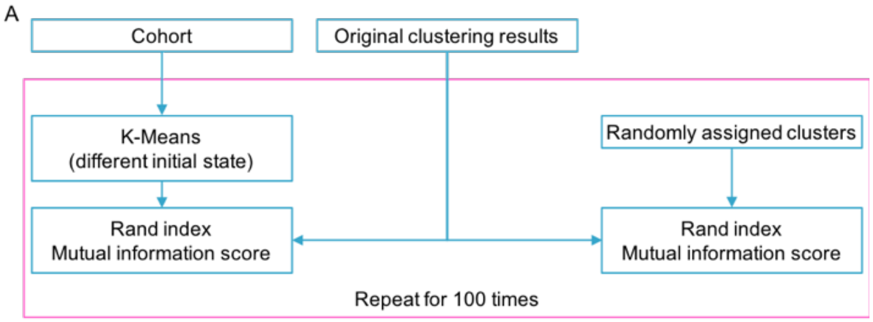

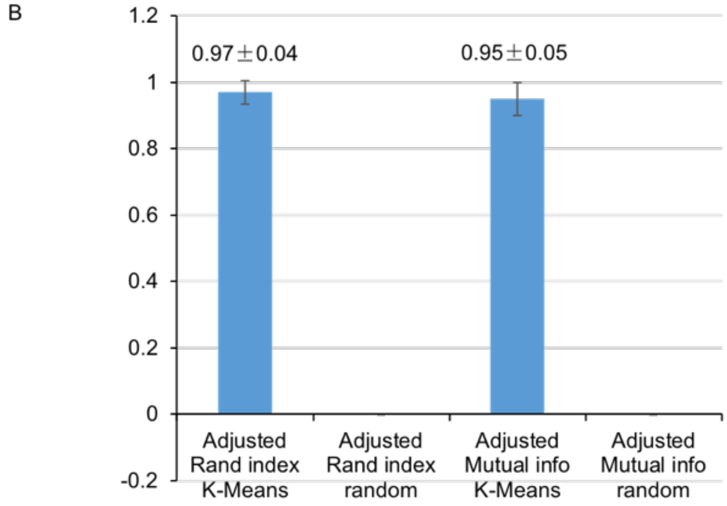

Extended Figure 4. Clustering stability test. (A) The workflow of K-means clustering stability test. (B) The adjusted rand index (ARI) and adjusted mutual information (AMI) among the 100 repeats showed high stability of the clustering results. The averages and standard deviations are shown in the bar plot.

References:

1. Hubert, L., and Arabie, P. (1985). Comparing partitions. *Journal of Classification 2*, 193-218.

2. Vinh NX, E.J., Bailey J (2010). Information theoretic measures for clustering comparison: variants, properties, normalization and correction for chance. *Journal of Machine Learning Research* 11, 2837-2854.

12. The nature of the clinical variables used is not entirely clear. In particular, it is stated on Page 9 that "we obtained 112 variables (including the derived ones). A detailed description for all the variables can be found in the supplemental methods section"; however, the exact figure/table listing these 112 variables does not appear to be present in the supplementary material. The closest appears to be S1 Table, which lists the abbreviation of some of the variables. A complete list and description of these variables might be added.

Response: We have updated all details for 112 variables in **S1 Table**. In this study, we included in total 112 variables as these variables are commonly measured and used in the cardio-oncology clinical practices implemented by both oncologists and cardiologists at the Cleveland Clinic Cardio-Oncology Center.

13. While the greater interpretability of a network-based approach is cited as a motivation for using a network rather than other machine learning techniques, relatively-interpretable statistical methods such as logistic regression would appear appropriate for identifying potential biomarkers/clinically actionable variables. The authors might consider confirming the significance of the network-discovered biomarkers/variables independent of subgroup analysis (e.g. with hazard ratios).

Response: We thank the Reviewer for this insightful suggestion. We have performed new Cox-regression analyses to validate the clinical variables with degree bigger than 10 in the clinical variable networks (**Figure 4** and **5,** revised **S8 Table**). The hazard ratio (HR) analysis results were consistent with our original findings (**Fig 5**) that NT-proBNP and Troponin-T are actionable biomarkers for cardiac subgroup. To be specific, NT-proBNP and Troponin-T were significantly associated with the increased risk of *de novo* CTRCD in *orange* subgroup (C1; NT-proBNP, HR = 1.362, p = 0.010; Troponin-T, HR = 1.139, p= 0.049, revised **S8 Table**). Meanwhile, the decreased LVEF (Left Ventricular

Ejection Fraction, parameter from echocardiogram) was associated with the increased risk of *de novo* CTRCD in *orange* subgroup (HR = 0.956, p = 0.003, revised **S8 Table**).

We also found that several clinical variables were significantly associated with survival outcomes in *pink* subgroup (highest mortality subgroup, revised **S8 Table**). For example, NT-proBNP is associated with poor survival outcome in *pink* subgroup (C4; HR = 1.374, p = 0.044). We have added these new findings in page 20 of the revised manuscript.

14. The CTRCD outcome currently consists of five cardiac conditions/events lumped together. It could be interesting to comment on the efficacy of the risk stratification on each of these conditions.

Response: We have computed the cumulative hazard and distributed percentage across five cardiovascular events separately: (heart failure) HF, (atrial fibrillation) AF, (coronary artery disease) CAD, (myocardial infarction) MI, and stroke (**Extended Figure 5** referring to revised **S5 Fig**). Specifically, the *orange* subgroup has highest cumulative hazard of *de novo* HF and *de novo* AF (p < 0.0001, **S5A Fig**), while the *blue* subgroup has lowest cumulative hazard of *de novo* HF, *de novo* AF, *de novo* CAD and *de novo* MI (p < 0.0001, **Extended Figure 5** referring to revised **S5 Fig**). The cumulative hazard of *de novo* stroke is slightly separated across 4 patient clusters (p = 0.055). One possible explanation is high disease heterogeneity of stroke. We have added these new findings in the revised manuscript.

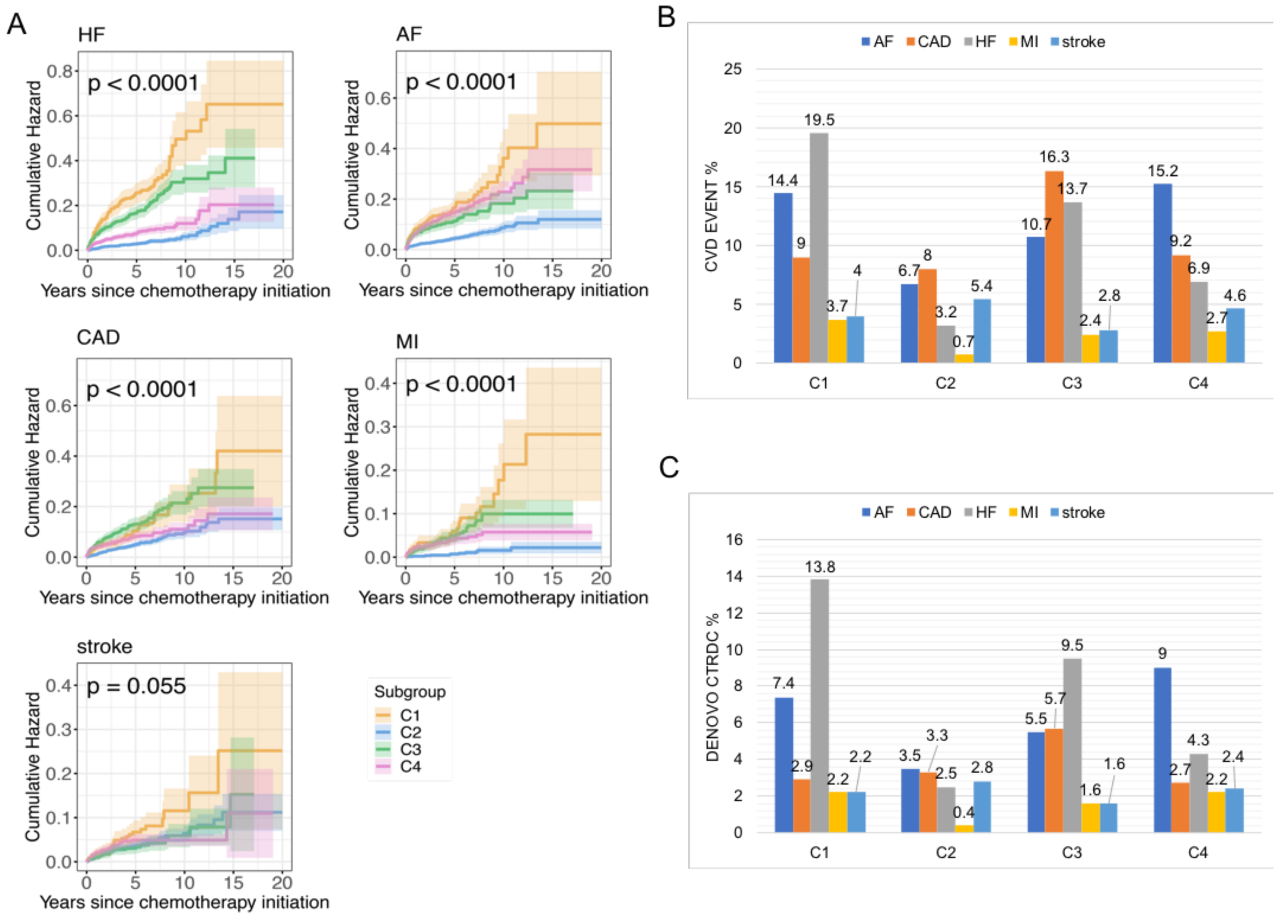

Extended Figure 5. Performance of the risk stratification on each CVD event. (**A**) Cumulative hazard of 5 *de novo* CVD events across 4 subgroups are shown. The log-rank test with the Benjamini & Hochberg (BH) adjustment was used for comparing the cumulative hazard among 4 subgroups. The shadow represents 95% confidence interval. (**B**) The percentage of 5 CVD events across four subgroups. (**C**) The percentage of 5 *de novo* CVD events (the patient has at least one type of cardiovascular events (heart failure, atrial fibrillation, coronary artery disease, myocardial infarction, or stroke) diagnosed after cancer therapy) across four subgroups.

15. There remain some minor grammatical issues [e.g. "...each patient totally had 56 clinical variables" (Page 8), "...from all cause" (Page 12) "...shows the worse mortality" (end of Page 15), "...have the limited clinical variables" (Page 19), "Total cohort were 4.532 patients" (Page 31)] and possible spelling issues [e.g. "K-mean" instead of "K-means" (Page 3/18)]

Response: We have checked the K-means spelling and the corrected term is K-means. We have revised all grammatical issues by our native speakers.

Responses to Reviewer #2

1. Authors aimed to perform unbiased cardiac risk stratification for cancer patients using a single institutional electronic medical record (EMR). **The study covers an interesting issue**, however there are several important concerns. In this study, the cardiovascular (CV) event and mortality were confirmed using their own EMR system. How did authors analyze the outcomes for patients who were initially treated in Cleveland Clinic but moved to another institution? What is the percentage of patients whose CV events or deaths are unknown for over six months?

Response: This cohort of patients are often seen by both oncologists and cardiologists at Cleveland Clinic and regularly followed up. For the very few cases that moved to another institution, the electronic medical records (EMRs) at Cleveland Clinic is a part of the Care Everywhere Network, which is used in 373 institutions across 48 states in the U.S. and allowed us see the details of visits from any such institution and therefore analyze relevant outcomes for these patients.

In addition, the percentage of patients whose CV events or deaths are unknown for over six months is **0%** as all patient charts were reviewed in details in order to assign a specific well-documented follow-up / alive date. We have added new explanations in the new revised manuscript.

2. Validity of the current study significantly relies on the quality of CV event verification, specifically the accuracy of their ICD codes. Is there any external validation study for their ICD code based diagnosis for atrial fibrillation, coronary artery disease, heart failure, myocardial infarction and stroke?

Response: In our study, we independently adjudicated each cardiovascular diagnosis (atrial fibrillation, coronary artery disease, heart failure, myocardial infarction and stroke) by at least two cardiologists to verify its accuracy. We didn’t perform external validation

study for ICD codes as there is lack of gold-standard external validation sets for ICD codes. We have discussed the limitation of possible false positive rate using ICD codes in the revised Discussion section (page 22 of the revised manuscript).

3. Lack of external validation for the current classification is an important limitation. If external validation is not possible, the whole study population could be Phenotype based classification without independent validation poses a significant potential for bias.
Response: We have performed four new subgroup analyses by randomly dividing into derivation cohorts for internal validation. As shown in the **extended Figures 2** and **3**, we re-identified four clusters which were well validated by survival and cardiovascular outcomes across four new patient clusters (subgroups) during internal validations. Please see detailed responses to Comment #10 of the Reviewer #1. We have added new findings of the internal validations in page 23 of the revised manuscript.

4. Not only the types and stages of specific cancer, but treatment information such as cumulative doses of anthracycline, radiation therapy or specific targeted agent confer a higher cardiotoxic effect. However, current risk stratification model does not include any information regarding types, stages or treatment of specific cancer.
Response: We thank the Reviewer for this good point. We have performed new analyses by adding cancer types, tumor stages and treatment information as new variables. We found the same results (**Extended Figure 6** referring to revised **S10 Fig**) compared to our original findings (**Fig 2B** and **Fig 2C)**. These new analyses revealed that confounding factors (i.e., tumor types, tumor stages or anticancer medications) have minor impacts on our patient network-based K-means clustering methodology. We have added new findings and explanations in the revised manuscript.

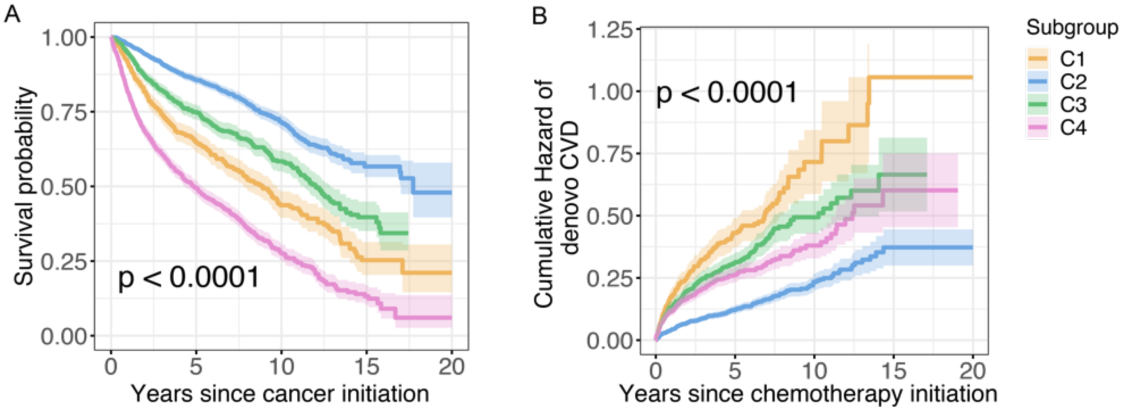

Extended Figure 6. Outcome validation for risk stratification model on the clinically-derived variables plus cancer type, cancer stage and treatment type. We re-identified four patient subgroups (clusters) using K-means clusters of patient-patient networks by adding cancer type, cancer stage and treatment types as new variables. (**A**) Kaplan-Meier curves to estimate all survival probability and **(B)** Cumulative hazard of *de novo* CTRCD (the patient has at least one type of cardiac event diagnosed after cancer therapy) across 4 subgroups are shown. The log-rank test with the Benjamini & Hochberg (BH) adjustment was used for comparing the cumulative hazard among 4 subgroups. The shadow represents 95% confidence interval.

5. Authors should mention about concrete application methods of their findings. If a new cancer patient is referred to cardio-oncology clinic, how can this risk stratification method can be applied?

Response: We thank the Reviewer for this insightful comment. To permit an unbiased risk stratification for new individuals, the clinical variables of individual will be collected by Redcap during the cardio-oncology practices. All collected variables will be normalized using a z-score measure for each variable. We can calculate the Euclidean distance (*d*) from the patient's vector of normalized variables to the center of each of the patient clusters. The patient can be assigned to a cluster/subgroup by network-distance measure (including shortest, closest, kernel, and center distance) in the patient-patient similarity network. The risk score for each patient will be computed based on his/her assigned cluster as determined by network proximity measure.

Our team are actively developing an online risk calculator by integrating all patient-patient network models in our cardio-oncology practices at the Cleveland Clinic. Due to the regulatory issues and the new patient safety policies implemented by COVID-19 pandemic, we cannot make our online risk calculator available in a short period during this manuscript revision. Your comments will motivate our future work and we have added new explanation in pages 23 and 24 of the revised manuscript.

Responses to Reviewer #3

Hou et al. have performed cardiac risk stratification for cancer patients using patient-patient network analysis. The field of cardio-oncology is emerging rapidly and creating a risk stratification of the patients in risk for developing cardiotoxicity is extremely important as it may allow the beginning of cardioprotective therapy and prevent the interruption of cancer therapy. **The use of network-based methodology is interesting and innovative.** I have some minor comments:

Response: We thank the Reviewer for the great summary and their support.

1. The term CTRCD is usually accepted for LVEF reduction >10% according to the ESC / ASE/ EACVI..... For my understating in this paper CTRCD was considered as AF / MI/ STROKE/ HF/ CAD, which may be confusing. Therefore, I would consider to change the term CTRCD to cardiovascular events.

Response: We agreed with the Reviewer that the term CTRCD is usually accepted for LVEF reduction >10%. In the original manuscript, we used both cardiovascular events and CTRCD. We used the *de novo* CTRCD term when this specific cardiovascular event (AF / MI / STROKE / HF / CAD) were occurred after cancer therapy initiation. We have added more explanation in the revised manuscript.

2. Do you have any information regarding the incidence of each event for the cardiovascular events? AF ? MI ?....

Response: We have re-calculated the incidence of 5 *de novo* CVD events from chemotherapy initiation date from 1 to 20 years (**Extended Table 1** referring to revised **S6 Table**). We found that 32% (1-year incidence is 6.11%) of *de novo* CVD events in first year after chemotherapy, especially for 35% of HF events (1-year incidence is 2.05%) and 36% of AF (incidence is 2.12%) (**Extended Table 1** referring to revised **S6 Table** and **Extended Figure 7** referring to revised **S7 Fig**). Notably, the 5-year incidence of total 5 *de novo* CVD events are 13.49 % (**Extended Table 1** referring to revised **S6 Table**). And 71 % of cardiac events occurred within the 5 years (**Extended Figure 7** referring to revised **S7 Fig**) during total 20-years' time window. We have added these new findings in page 18 of the revised manuscript.

Extended Table 1 The incidence of 5 *de novo* CVD events from chemotherapy initiation 1 year, 5 years, 10 years and 20 years.

	**HF %**	**AF %**	**CAD %**	**Stroke %**	**MI %**	**Total CVD events %**
**1-year**	2.05	2.12	0.89	0.67	0.39	6.11
**5-years**	4.34	4.17	2.33	1.64	1.01	13.49
**10-years**	5.40	5.35	3.28	2.05	1.25	17.34
**20-years**	5.94	5.92	3.56	2.35	1.36	19.13

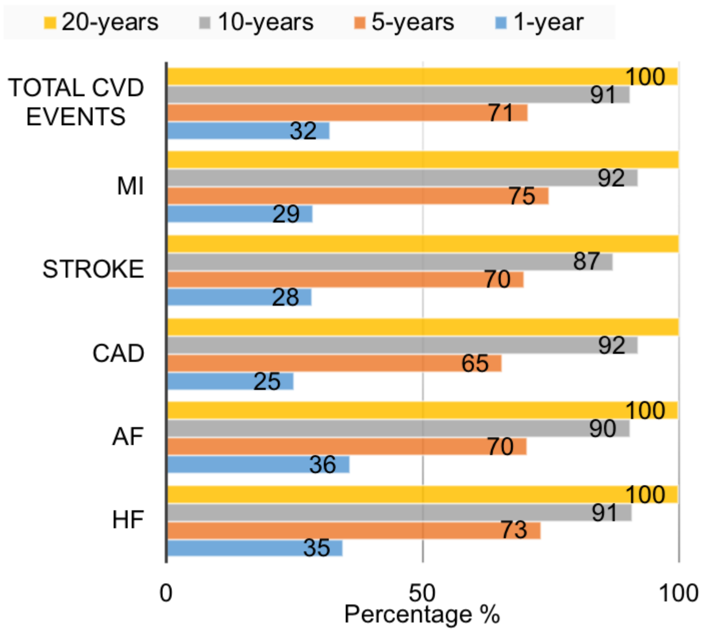

Extended Figure 7. The cumulative percentage of 5 *de novo* CVD events change during 1 to 20 years after cancer therapy.

3. Can you explain why you choose to include AF with MI/STROKE/HF/CAD? The incidence and the severity of AF is not equivalent to the other events and I'm not sure that should be included in the same category. Most probably cancer therapy will not be interacted due to AF as it will be due to MI or HF.

Response: Although AF does not usually interrupt cancer therapy, there is evidence that the presence of AF is strongly associated with a worse prognosis in patients with cancer (Aspberg et al, *Journal of Stroke and Cerebrovascular Diseases* 2020; Fitzpatrick et al, *Thrombosis Research* 2017). We therefore included AF in our original study as well. In a recent study, a group reported an AF HR greater than for stroke in cancer patients: atrial fibrillation (HR 1.30, 95% CI 1.09-1.53) and stroke (HR 1.21, 95% CI 1.11-1.32) (Liu et al, *Aging* 2019).

References:

1. Aspberg S, Yu L, Gigante B, Smedby KE, Singer DE. (2020) Risk of ischemic stroke and major bleeding in patients with atrial fibrillation and cancer. *Journal of Stroke and Cerebrovascular Diseases* 29:104560.
2. Fitzpatrick T, Carrier M, Le Gal G. (2017) Cancer, atrial fibrillation, and stroke. *Thrombosis Research* 155:101-105.
3. Liu D, Ma Z, Yang J, Zhao M, Ao H, Zheng X, Wen Q, Yang Y, You J, Qiao S, Yuan J. (2019) Prevalence and prognosis significance of cardiovascular disease in cancer patients: A population-based study. *Aging* 11:7948-7960.

4. The outcome of the paper included cardiovascular events and all-cause mortality. Do you have any information regarding CV mortality which is more relevant to cardiotoxicity since probably the majority of the death were due to cancer reasons and not relates to cardiotoxicity.

Response: Although we have all-cause mortality, we were hoping to also define cardiovascular mortality in this cohort in our original study. Cardiovascular mortality was adjudicated in patients that died in hospital however adjudication of cardiovascular mortality in those that died outside of hospital was not possible. Another thought was to

try and obtain death certificates for cause of death, however this leads to methodological concerns due to reported inaccuracies in death certificates (Lloyd et al, *Annals of Internal Medicine* 1998; Smith et al, *Archives of Internal Medicine* 2001). In summary, the confirmed CV mortality in our dataset is very low and we are not able to perform CV mortality analysis by under-power issue.

References:

1. Lloyd-Jones DM, Martin DO, Larson MG, Levy D. (1998) Accuracy of death certificates for coding coronary heart disease as the cause of death. *Annals of Internal Medicine* 129:1020-1026.
2. Smith Sehdev AE, Hutchins GM. (2001) Problems with proper completion and accuracy of the cause-of-death statement. *Archives of Internal Medicine* 161:277-284.

5. Regarding the observation according to years - I'm not sure that we can conclude that the high mortality in 5 years imply the dose time dependent since cancer patients has low 5 years survival, and furthermore the number of patients followed after 10 years is very low.

Response: We thank the Reviewer for this critique. We have removed “the dose time dependent cardiotoxicity” and added more explanations in the revised manuscript.
