# Supplementary material for: Cardiac risk stratification in cancer patients: A longitudinal patient–patient network analysis
Source: PLoS Med. 2021 Aug 2;18(8):e1003736. doi: 10.1371/journal.pmed.1003736 (PMC8366997; doi:10.1371/journal.pmed.1003736)
Supplement: Attachment — Submitted filename: Response_Letter_Dr.Cheng.pdf [file pmed.1003736.s031.pdf]

Manuscript #: PMEDICINE-D-20-00473R2

Responses to Reviewer #1:

Reviewer #1: We thank the authors for addressing most of the points raised in the previous review round.

1. On the psnCVD clustering, there might have been slight confusion on the "actual" network after clustering that is used for assigning patients (supposedly using all 4632 patients, described in the Network clustering section), and the "visualization" network that has some less-similar patients removed (and which has minimized network density). To the best of our updated understanding, the "visualization" network does not actually have any impact on the main results as reported in the Network-based discovery of novel cardiac risk subgroups section; these subgroup results are obtained from the "actual" psnCVD, which moreover can assign a subgroup to any new patient (after that patient's network profile is computed), even if the new patient is actually dissimilar to almost all other patients. As such, the psnCVD can always assign a new patient (possibly from another source/hospital) to a subgroup, given that patient's data. If this is correct, the authors might consider emphasizing this interpretation, since it was not entirely clear that the visualization density minimization procedure does not actually have an impact on the results.

Response: We thank the Reviewer for this great summary. Yes, all patients are predicted and assigned by the psnCVD. The "visualization" network does not actually have an impact on discovering novel cardiac risk subgroups. The density minimization procedure is a network layout approach to visually illustrate the predictions from the psnCVD. We have added more explanations in the revised manuscript and figure legend to clarify it. Specifically, we have added more explanation that the density minimization procedure was only used to visualize the network to avoid a hairball network visualization in the revised manuscript. Although the density minimization procedure doesn’t have a direct impact on improving performance of the psnCVD, it can optimize network visualization for new patients when predicting new patients in a

specific subgroup to help patients better understand their subgroup distribution visually (network layout).

In addition, as the Reviewer pointed out that a new patient with known network profile can be assigned a subgroup, we have adopted this strategy and performed additional experiments to validate the generalizability of our model (see response to your Comments #4).

2. Under the Network clustering section, it is stated that "we instead examining the survival analysis and cardiovascular outcome analyses at different number of clusters. The highest number that produced clusters with distinguishable survival and cardiovascular outcome was 4". How was "distinguishable" determined in this case, i.e. was there some quantifiable metric, or was it by observation?

Response: In this study, we chose the best cluster number (K=4) using subject matter expertise based on a combination of factors: (i) significantly distinguishable survival rate and (ii) cardiovascular outcome by Kaplan-Meier (KM) estimator with Log-rank test (Log-rank $p < 0.05$, **Extended Figure 1 [S1 Fig] and S2 Table**); and (iii) the highest number of clusters to identify more new patient subgroups. We did not choose K=3 as we pursue to identify more novel patient clusters or subgroups. We have added the detailed explanation in page 11 of the revised manuscript.

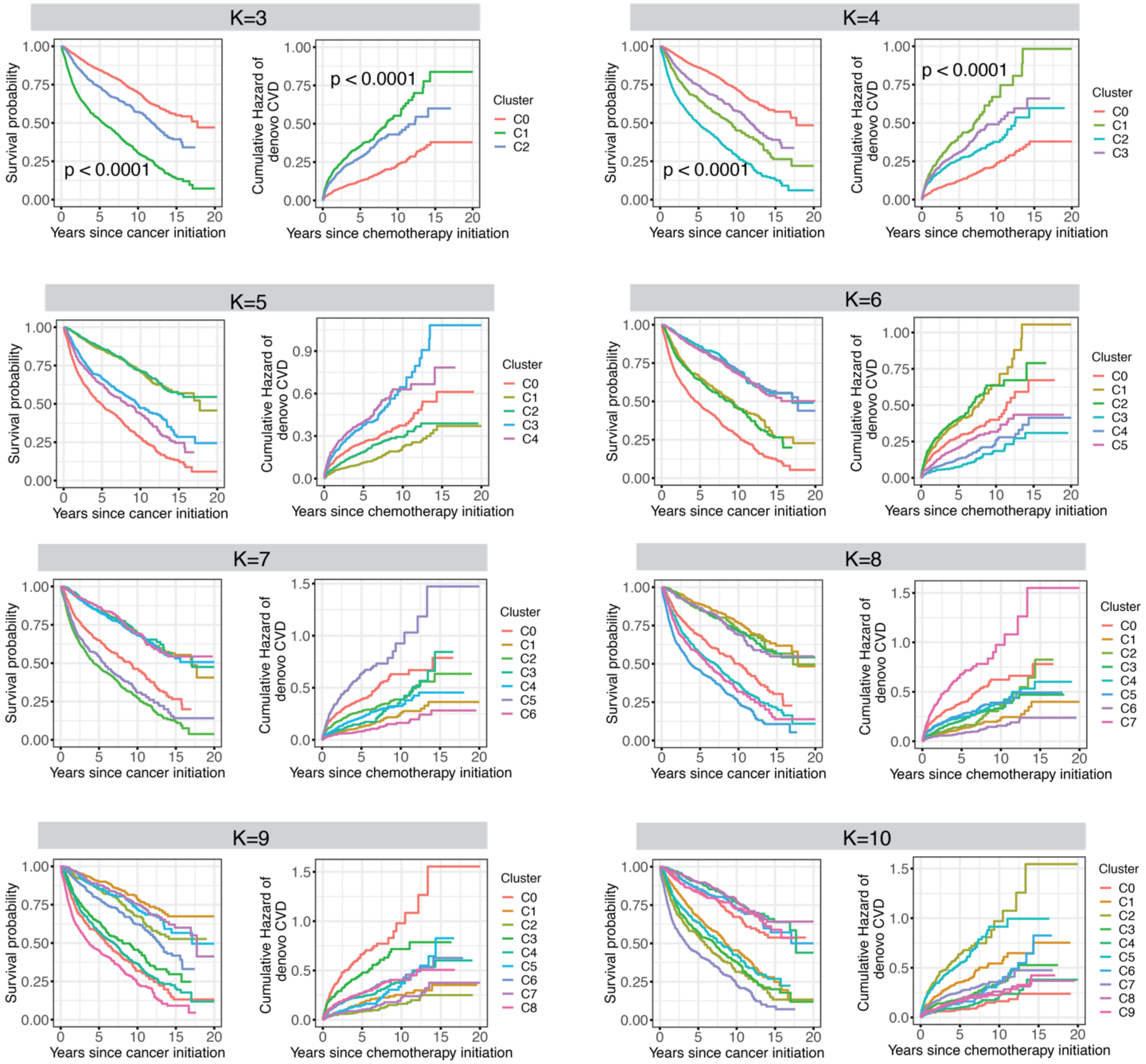

Extended Figure 1. Survival and cardiovascular outcome for different number of clusters using Kaplan-Meier analysis. The number of clusters (K) from 3 to 10 were tested. The Log-rank test were used to evaluate the statistical significance. K=4 resulted in the highest number of clusters with distinguishable survival and cardiovascular outcome. All pairwise p values between the subgroups for each K value were summarized in **S2 Table.**

3. It would be helpful if the main characteristics of the four discovered subgroups (as described in detail in the Network-based discovery of clinically actionable variables section) be summarized within a table.

Response: We thank the Reviewer for this great point. We have summarized the main characteristics of clinically actionable variables across four network-predicted patient subgroups in the revised **S10 Table**, including mean value with 95% CIs, one-way ANOVA statistics, two network properties (degree and centrality), and others.

4. The lack of an independent validation cohort remains a major concern, though this has been mitigated to an extent by two internal validation approaches. More details might be provided on what was done for these internal validation approaches, though: **For 1)**, the subgroups appear to be split by follow-up time windows (1997-2012), (1997-2015), (1997-2017); was the approach used some form of cross-validation (e.g. to cluster patients from subgroup (1997-2017), the psnCVD was constructed based on the other two subgroups)? **For 2)**, similarly, given a sampling ratio of 50%, was the psnCVD constructed based on 50% of the randomly-split data, and then evaluated on the remaining 50% to produce the charts?

Response: We thank the Reviewer for the agreement that we can use two internal validation approaches to replace the external validation by lack of external patient data in the Cardio-Oncology Research Community. In the previous revision, we split the data by time/random, then applied the method separately for the split data sets to see if the same patterns can still be observed in two sub-cohorts. These experiments were not in the form of cross-validation. As the Reviewer pointed out, a new patient with known network profile can be assigned a subgroup. We apologize that we did not adopt such strategy earlier to further show the robustness of our method. Therefore, we have conducted new random-split and time-split training-test validation experiments.

Here, we split the data by time and by randomization to select one set as the "training" set and the other as the "test" set. In the time-split experiment, the set with earlier time was regarded as the training set. To be specific, we fitted the K-means model on the training set, and used the model to predict the clusters for new patients in the test set. The detailed diagram of the new experiments is illustrated in **Extended Figure 2 (**referring to revised **S13 Fig)**. The results for random-split and time-split training-test validation can be found in **Extended Figure 3 (**referring to revised **S14 Fig)** and **Extended Figure 4 (**referring to revised **S15 Fig)**, respectively.

In **Extended Figure 3** (to answer question 1), the patients were split by three different time points to generate three training/test pairs: the training and test sizes are 50% versus 50%, 60% versus 40%, and 80% versus 20%. In **Extended Figure 4** (to answer question 2), all patients were randomly split to two equally sized sets by three random experiments.

As shown in **Extended Figures 3 and 4**, the patients in the test sets have statistically significantly (Log-rank test, $p < 0.05$) distinguishable survival and *de novo* CVD risk patterns among the groups, and the patterns are consistent with the training set groups. These results suggest a high generalizability of our methodology in the real-world application settings. We agree with the Reviewer that an independent validation cohort is still needed in the future. We have added these new findings and more explanations about future independent cohort validation on pages 22-23 of the revised manuscript.

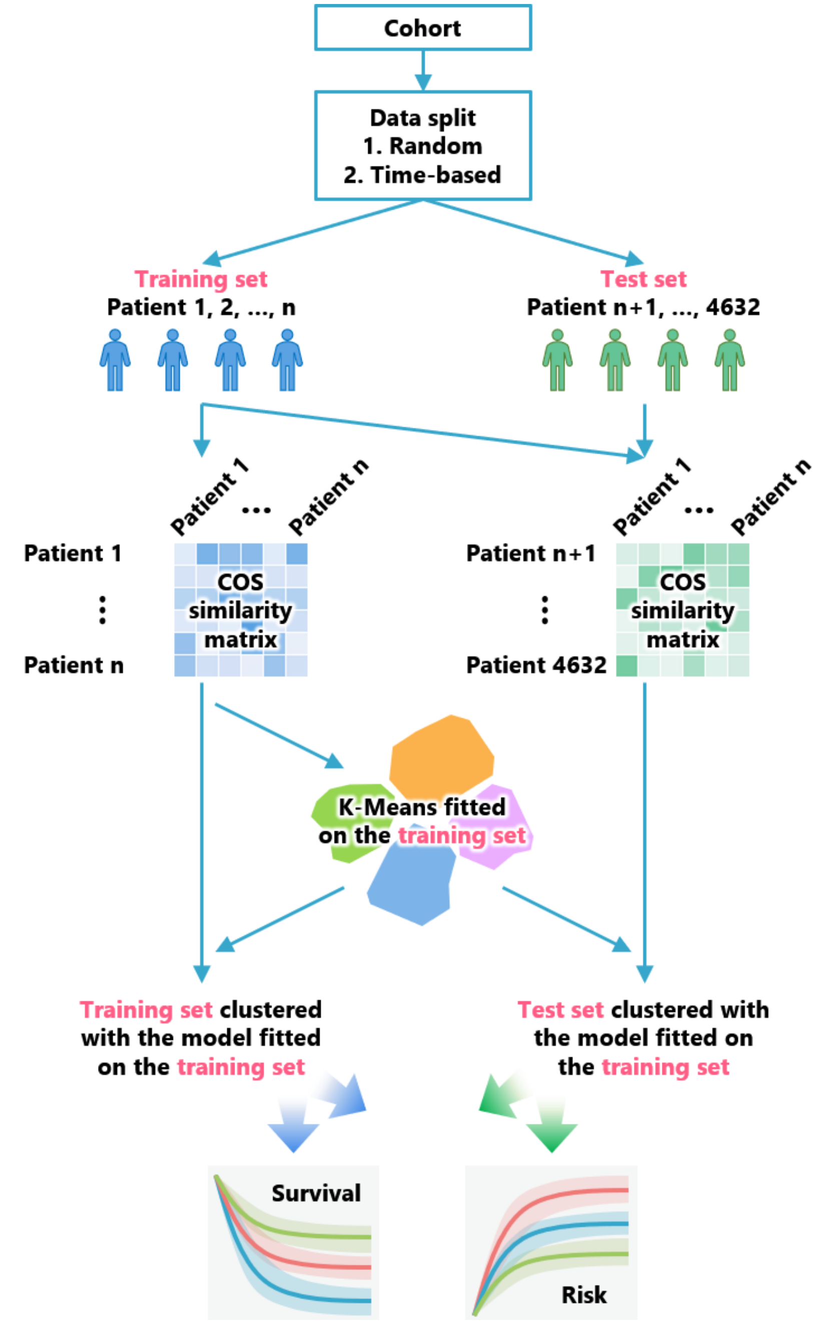

Extended Figure 2. The workflow of the training-test validation strategy to test the generalizability of psnCVD models. All patients were split randomly or by time to training and test sets. We computed the cosine similarity matrix for the training set patients (blue matrix) and for the test patients (green matrix) against the training patients. Next, K-means clustering was performed on the training set and was used to predict both the training and test sets. The predicted clusters were evaluated for the survival and *de novo* CTRCD risk for both the training and test sets.

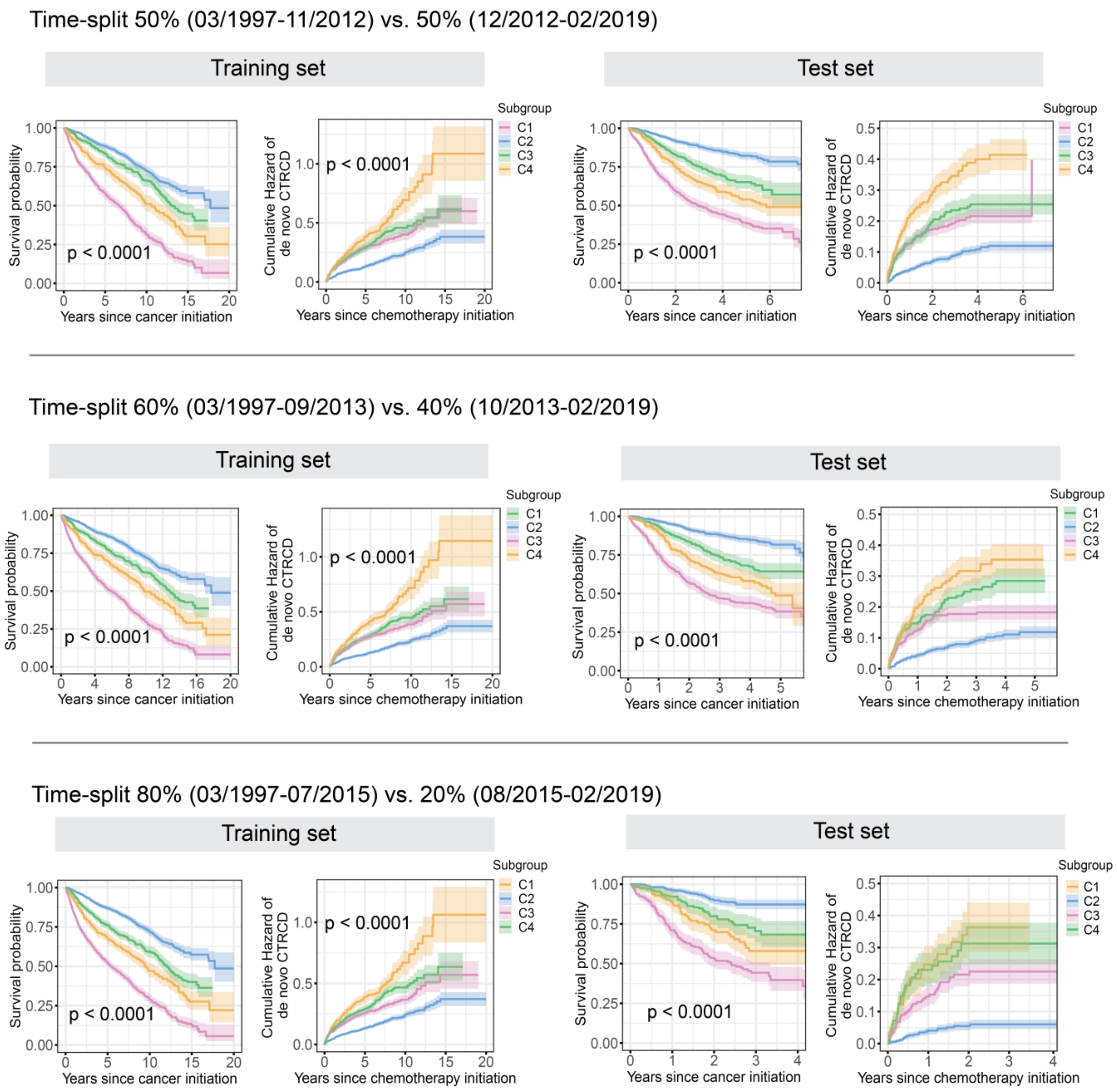

Extended Figure 3. Evaluation of the generalizability of psnCVD models using time split cohorts. Patients were split by their cancer diagnosis time to three training set / test set pairs: 50% versus 50%, 60% versus 40%, and 80% versus 20%, respectively. The survival probability and cumulative hazard of *de novo* CVD of the training sets and test sets were evaluated. Log-rank tests show statistically significant difference in both survival probability and cumulative hazard of *de novo* CTRCD for the patient groups in the test sets, consistent with that of the training sets.

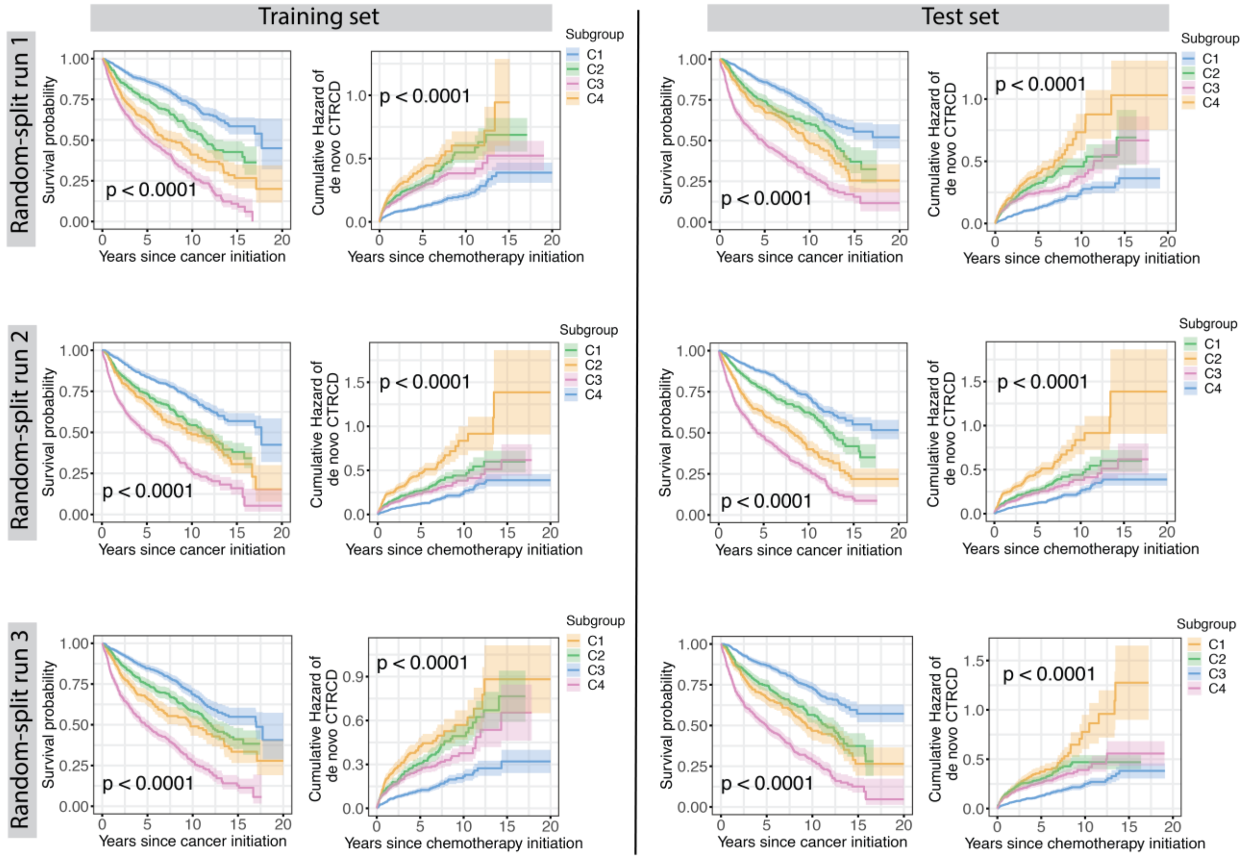

Extended Figure 4. Evaluation of the generalizability of psnCVD models using randomly split cohorts. The survival probability and cumulative hazard of de novo CTRCD of the training set (50%) and test set (50%) were evaluated in three independent random experiments. Log-rank tests show statistically significant difference in survival probability and cumulative hazard of *de novo* CTRCD for the patient groups in the test sets, consistent with that of the training sets.

5. There remain some minor grammatical issues, e.g.

Page 10: "we instead examining the survival analysis..."

Page 11: "we repeated 100 times of the K-means clustering analyses..."

Page 14: "Totally, 1,670 (36%) of patients have at least one type of diagnosed cardiac events..."

Page 17: "long-time exposure..." (long-term?)

Response: We have revised all grammatical issues and the text was double checked by native speakers.

Responses to the Academic Editor:

One issue that I didn't see raised by any of the other reviewers is the overall model performance. If I understand correctly the authors divide their work into three main components: clustering, risk prediction, and a sort of causal inference to discover modifiable risk factors. The first bit is strong. The second component (risk prediction) is done in each of the main clusters separately and HRs compared. Normally, what we would like to also see is the overall model performance (e.g., AUROC/AUPRC) in particular in comparison with conventional models. So, does the modelling add much to a simple Cox model that is applied to all predictors (paying attention to collinearity etc as we would usually do)?

Response: We thank the Academic Editor for these great points. We agree that it is very important to evaluate the overall model performance using AUROC/AUPRC. However, we should emphasize that (1) the clustering part of our method was unsupervised-based, and (2) one of the main goals of our study was to identify novel patient subgroups that exhibit distinguishable clinical outcomes and *de novo* cardio-toxicty. Therefore, our models are not comparable to Cox models or other conventional (supervised) machine learning models. However, we used conventional methods, such as Kaplan-Meier analysis to validate the patient subgroup findings to show distinct clinical outcomes (survival and *de novo* CVD risk). Our results indeed show that the novel patient groups have statistically significant distinguishable outcomes. In addition, although we cannot directly evaluate the patient subgroups using metrics such as AUROC/AUPRC for our unsupervised-based models, we adopted a training-test strategy to evaluate the generalizability of our models (see Response to the Comments #4 of the Reviewer #1).

To respond the Editor's concerns, we performed new time-dependent AUROC (Uno, *et al.*, *Journal of the American Statistical Association* 2007; Hung and Chiang, *Canadian Journal of Statistics* 2010; Kamarudin, *et al.*, *BMC Medical Research Methodology* 2017) analysis. As shown in **Extended Figure 5A,** the overall AUROC of Cox proportional hazard models ranges from 0.79 to 0.85 for test sets across different time-point cohorts. In addition, we found that our psnCVD models can further improve performance of the Cox proportional hazard models (**Extended Figure 5B-D [**referring

to the revised **S16 Fig]**). We have added these new findings and more explanations in the revised manuscript.

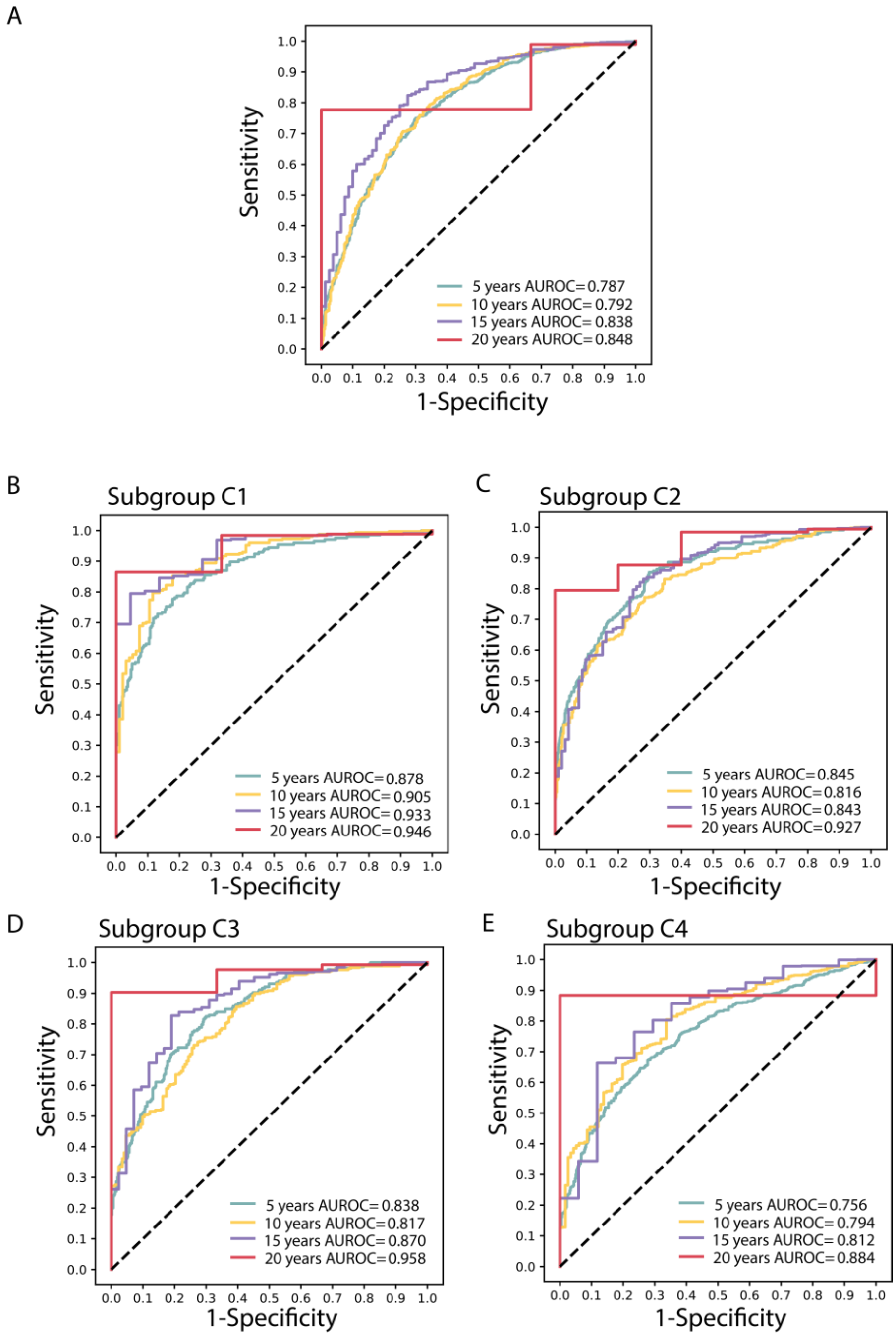

Extended Figure 5. The performance of Cox proportional hazard model using the entire cohort (A) and individual patient subgroups identified by psnCVD models (B-E). For each subplot, all patients (**A**) or patients in individual subgroups (**B-E**) were split to training (50%) and test (50%) set. The clusters for the patients in the test set were predicted based on the model

fitted on the training set. Time-dependent AUROC was used to evaluate the model performance of the test sets.

We agree with the Academic Editor that collinearity might influence the performance of the models. To inspect collinearity, we computed the Pearson Correlation Coefficient (PCC) among all the features. As shown in **Extended Figure 6 (**referring to revised **S12 Fig)**, the |PCC| value is less than 0.25 among numerical variables, and 95% |PCC| values are < 0.25, suggesting overall low collinearity of the variables. We have added these new data and more discussion about the influence of collinearity in the revised manuscript.

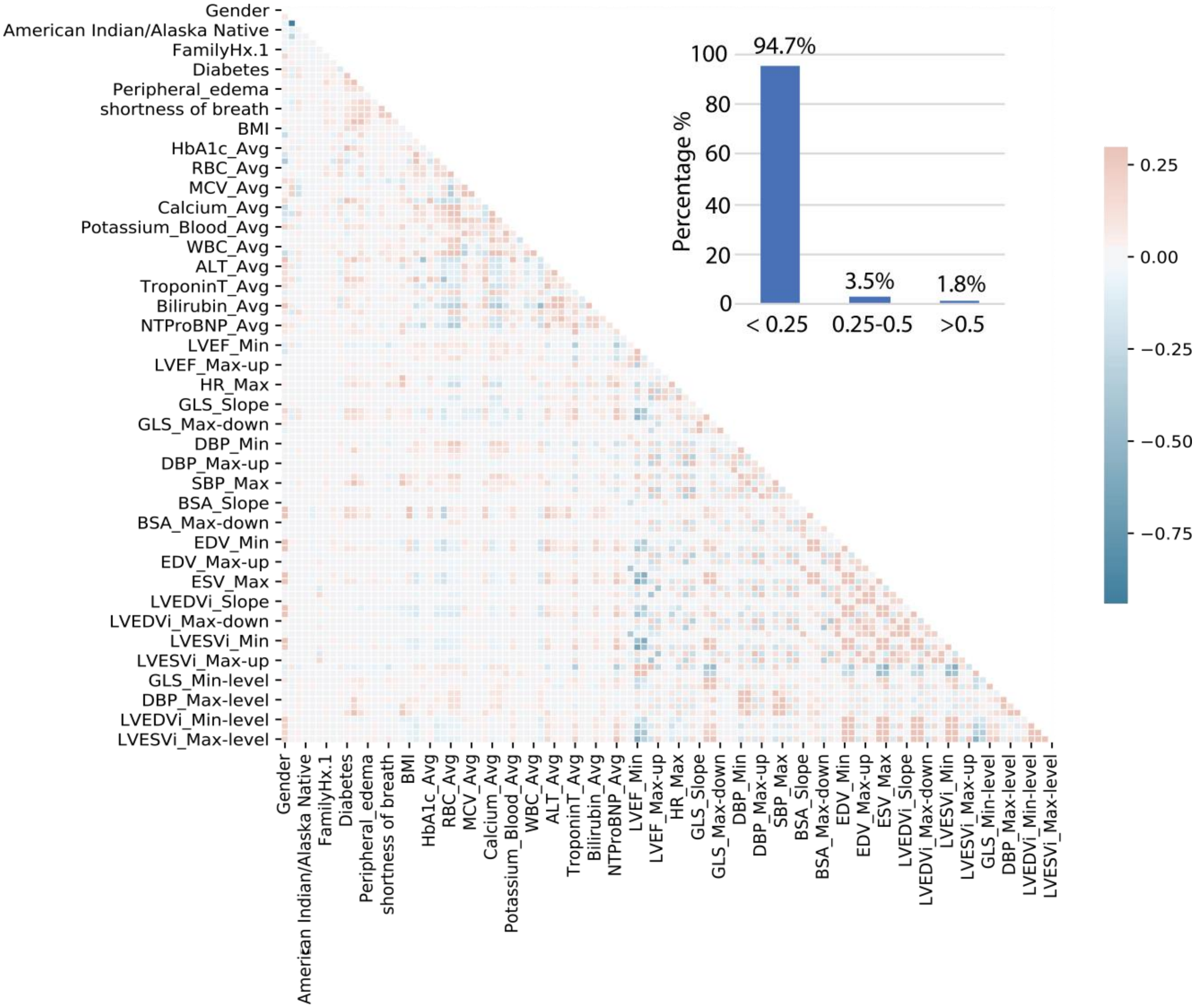

Extended Figure 6. Pairwise Pearson Correlations (PCC) among the used 112 clinical variables. The gradient red color means positive correlation and gradient blue color means

negative correlation. The 112 labels showed in **S1 Table**, and the order were same with the order in heatmap.

References:

1. Uno H, Cai T, Tian L, Wei LJ. Evaluating Prediction Rules for t-Year Survivors With Censored Regression Models. *Journal of the American Statistical Association* 2007; 102: 527-537.
2. Hung H, Chiang C-T. Estimation methods for time-dependent AUC models with survival data. *Canadian Journal of Statistics* 2010; 38: 8-26.
3. Kamarudin AN, Cox T, Kolamunnage-Dona R. Time-dependent ROC curve analysis in medical research: current methods and applications. *BMC Medical Research Methodology* 2017; 17: 53.

The comparison of HRs does not suggest that the clusters are hugely different in risk patterns and the 'unbiased' discovery of modifiable risk factors does not lead to any surprises. Please compare the performance of the predictive model with a standard regression/hazard model.

Response: To test whether the patient clusters have significantly different clinical outcomes, we computed the p values of pairwise subgroup comparisons using log-rank test. The results show that the survival and *de novo* CVD risk were significantly different among subgroups (**Extended Table 1** referring to revised **S2 Table, K=4**). We have added the comparisons of our method and conventional methods in **Extended Figure 5.**

We greatly appreciate the constructive comments and suggestions from the academic Editors. We have revised our manuscript and added additional results (**S1, S12-16 Figs**, and **S2 Table** [Extended Figure 1-6, Extended Table 1]). We believe our study has been significantly improved and will provide valuable information (both the methodology and the results) for the research community.

Extended Table 1. Pairwise subgroup comparisons for the results of survival and *de novo* CVD risk.

**p value (Log-rank test) of survival**			
subgroups	***C1***	***C2***	***C3***

C2	2.00E-16	-	-
C3	2.40E-05	2.00E-16	-
C4	8.10E-14	2.00E-16	2.00E-16
**p value (Log-rank test) of Cumulative Hazard of *de novo* CVD**			
subgroups	***C1***	***C2***	***C3***
C2	2.00E-16	-	-
C3	0.014	2.00E-16	-
C4	3.60E-06	5.80E-13	0.02

Responses to the Editors:

1- Did your study have a prospective protocol or analysis plan? Please state this (either way) early in the Methods section.

Response: Our study has a retrospective plan in place. The patient pool in this study represents oncology patients seen by oncology specialists at our institution undergoing cancer therapeutics and referred for cardiology evaluation/testing based upon cardiac risk factor profile or cardiac comorbidity. Once patients were identified, patient information was collected. We have added it in the revised Methods section (page 8).

The reasons for referral to the cardio-oncology service were for echocardiographic cardio-surveillance (“baseline and serial evaluation for patients on therapy with cardiotoxic agents”), and / or for pre-existing cardiovascular risk factor / disease management. All patients had baseline electrocardiogram and echocardiography performed. Follow-up cardiology studies were performed at the discretion of the cardio-oncology team. As per standard treatment protocols, patient’s vitals were checked and history and physical were obtained at every cancer therapy visit.

a) If a prospective analysis plan (from your funding proposal, IRB or other ethics committee submission, study protocol, or other planning document written before analyzing the data) was used in designing the study, please include the relevant prospectively written document with your revised manuscript as a Supporting

Information file to be published alongside your study, and cite it in the Methods section. A legend for this file should be included at the end of your manuscript.
b) If no such document exists, please make sure that the Methods section transparently describes when analyses were planned, and when/why any data-driven changes to analyses took place.
c) In either case, changes in the analysis-- including those made in response to peer review comments-- should be identified as such in the Methods section of the paper, with rationale.
Response: We selected ***b) No such document exists.*** All changes have been marked and added to the main manuscript document. Analysis were planned in 2019 and no data-driven changes to analyses took place.

2- Please ensure that the study is reported according to the STARD 2015 reporting guideline for diagnostic accuracy studies, and include the completed STARD checklist as Supporting Information. Please add the following statement, or similar, to the Methods: "This study is reported as per the STARD 2015 reporting guideline for diagnostic accuracy studies (S1 Checklist)."
The STARD guideline can be found here: http://www.equator-network.org/reporting-guidelines/stard/
If you feel a different checklist is more appropriate, please include that instead.
When completing the checklist, please use section and paragraph numbers, rather than page numbers.
Response: We thank the Editor for their comments. We have edited our manuscript to reflect the STARD guidelines. The following has been added to the manuscript: This study is reported as per the STARD 2015 reporting guideline for diagnostic accuracy studies (**S1 Checklist**).

3- Please revise your title according to PLOS Medicine's style. Your title must be nondeclarative and not a question. It should begin with main concept if possible. Please place the study design ("A randomized controlled trial," "A retrospective study," "A modelling study," etc.) in the subtitle (ie, after a colon).

Response: We have changed the title to "*A Longitudinal Patient-Patient Network Analysis of Cardiac Risk in 4,600 Cancer Patients from 1997 to 2019*".

4- In the Abstract Methods and Findings, please include the population and setting, and years during which the study took place.

Response: We have added the population and setting, and years of our study in the Abstract - Methods and Findings on page 3.

5- In the Abstract and throughout, please include p-values alongside 95% CIs for all comparisons.

Response: We thank the Editor's comments. We have added 95% CIs for all comparisons which we can obtain 95% CIs in the revised manuscript.

6- In the last sentence of the Abstract Methods and Findings section, please describe the main limitation(s) of the study's methodology.

Response: We thank the Editor for their comments. We have added the discussion of the main limitation in Abstract Methods and Findings section on page 3-4.

7- Please make sure all results are first reported in the Results section, rather than the Discussion, which should be focused on interpretation.

Response: We thank the Editor's suggestions. We have moved all results from Discussion to the revised Results section.
