# Supplementary material for: Cardiac risk stratification in cancer patients: A longitudinal patient–patient network analysis
Source: PLoS Med. 2021 Aug 2;18(8):e1003736. doi: 10.1371/journal.pmed.1003736 (PMC8366997; doi:10.1371/journal.pmed.1003736)
Supplement: Attachment — Submitted filename: Response_Letter_Cheng.pdf [file pmed.1003736.s032.pdf]

Responses to the Reviewers' and the Editors' comments

Manuscript #: PMEDICINE-D-20-00473R3

Responses to the Reviewer' comments:

1. We thank the authors for responding to our previous comments, and in particular the additional experiments presented towards validating robustness.
Response: We thank the Reviewer's support of our manuscript.

2. However, while the new S12 Fig is captioned as "Pairwise Pearson Correlations among the used 112 clinical variables", there appear only 38 variables shown in the figure. It is recognized that it may be impractical to display a full matrix of 112 labels, but in this case the caption of the figure might be updated, and commentary made about whether the displayed variables are representative.
Response: We thank the Reviewer's comments. We have re-ranked variables based on four variable categories from A to Z. Duo to the space limitation, the labels in heatmap show one variable name in every 3 variables. To respond the Reviewer's concern, we added a new **S10 Table** to illustrate the order of the entire correlation matrix of 112 variables, and the order of labels were same with the label ranked in the heatmap (**S12 Fig**).

Responses to the Editors' comments

1. Please adapt the title to better match journal style. We suggest: "Cardiac risk stratification in cancer patients: A longitudinal patient-patient network analysis".
Response: We have changed the title as "*Cardiac risk stratification in cancer patients: A longitudinal patient-patient network analysis*" in the revised manuscript.

2. We suggest substituting "echocardiogram" for "Echo" throughout.

Response: We have changed "echocardiogram" for "Echo" in revised manuscript and all figures (Fig1, Fig 4, Fig5, FigS11, FigS12) as well.

3. Please trim the "Background" subsection of your abstract, aiming to remove 1-2 sentences.

Response: We have shorten the "Background" in revised manuscript.

4. Please quote summary demographic characteristics for study participants in your abstract.

Response: We have added demographic characteristics for study participants in abstract.

5. We note that you quote some very small p values in the abstract. We generally ask for p values to be quoted exactly or as p<0.001, unless there is a specific statistical reason for very small values to be stated exactly. We are not aware of such reasons in this case, and ask you to observe this convention throughout the paper.

Response: We have changed p values as exact p values and used p<0.001 for the smaller p-values in the revised manuscript.

6. Please remove the sentence beginning "However, further prospective validation studies ..." from your abstract. You may wish to make this point as part of a new sentence addressing study limitations (see immediately below) or move it to the Discussion section.

Response: We have removed the sentence "However, further prospective validation studies ..." to limitations section.

7. Please add a new final sentence to the "Methods and findings" subsection of your abstract. This should begin "Study limitations include ..." or similar and should list 2-3 of the study's main limitations.

Response: We have added study's main limitations in "Methods and findings" subsection.

8. Please adapt the "Conclusions" subsection of your abstract to begin "In this study, we found that ..." or similar and adapt the tense(s) used as needed.

Response: We have revised it as the suggested format.

9. In the "Author summary", please add a few words to the first point of the "What did the researchers do ..." subsection to briefly describe your methodology.

Response: We have added a brief description of methodology in the "Author summary".

10. Please restructure the end of the Introduction section of your main text. The final paragraph should briefly state the aim of the study, but not summarize the conclusions.

Response: We have changed final paragraph in the revised manuscript.

11. Noting "... our [model] excels ..." we ask you to adapt the language used in places to avoid an impression of exaggeration.

Response: We have revised it in the entire manuscript.

12. Early in the Methods section (main text), you mention a "retrospective plan". Please adapt the wording here to state that there was no prespecified analysis plan (assuming this is the case - if not, please attach the plan or protocol as a supplementary document, referred to in the text). You may wish to note that the analyses were prespecified and that no data-driven changes were made.

Response: We have added "*Our retrospective study hasn't prespecified analysis plan*." in the revised Methods section.

13. Early in the Discussion section, we suggest rewording "patients' mortality and subsequent cardiac outcomes."

Response: We have revised it as "*the risk of cardiac dysfunction in cancer survivors*"

14. Throughout the text, please adapt reference call-outs to remove spaces from within the square brackets (e.g., "... ventricular dysfunction [15,16].").

Response: We have revised it in the whole manuscript.

15. Please use the general style "...4 categories ..." consistently throughout the paper, although numbers should be spelt out at the start of sentences.

Response: We have uniformed four categories by “general demographics, echocardiogram, lab testing, and cardiac” throughout the entire manuscript.

16. Please remove the information on data availability, funding and competing interests from the end of the main text. In the event of publication, this information will appear in the article metadata via entries in the submission form.

Response: We have removed it.

17. In table 1 and any other instances in the ms, please substitute "sex" for "gender" where appropriate.

Response: We have changed “gender” to “sex”.

18. Please rename the attached checklist "S1_STARD_Checklist" and refer to it by this label in the Methods section.

Response: We have renamed the checklist and cite it in Methods section.

19. Please adapt the checklist so that individual items are referred to by section (e.g., "Methods") and paragraph number, not by line or page numbers (which generally change upon publication).

Response: We have adapted the checklist by section (e.g., "Methods") and paragraph number.

20. "Label" is misspelt in S1 table.

Response: We have corrected this typo and double check the entire manuscript files.
